# Causal association of menstrual reproductive factors on the risk of osteoarthritis: A univariate and multivariate Mendelian randomization study

**DOI:** 10.1371/journal.pone.0307958

**Published:** 2024-08-30

**Authors:** Xinzhe Tan, Yifang Mei, Yihao Zhou, Zhichao Liao, Pengqi Zhang, Yichang Liu, Yixiao Han, Dongyan Wang

**Affiliations:** 1 College of Acu-moxibustion and Massage, Heilongjiang University Of Chinese Medicine, Haerbin, Heilongjiang Province, China; 2 Department of Rheumatology and Immunology, The Third People’s Hospital of Shenzhen, Shenzhen, Guangdong Province, China; 3 Department of Acupuncture and Moxibustion, Heilongjiang University of Chinese Medicine Affiliated Second Hospital, Haerbin, Heilongjiang Province, China; University College London, UNITED KINGDOM OF GREAT BRITAIN AND NORTHERN IRELAND

## Abstract

**Objective:**

Several observational studies have revealed a potential relationship between menstrual reproductive factors (MRF) and osteoarthritis (OA). However, the precise causal relationship remains elusive. This study performed Mendelian randomization (MR) to provide deeper insights into this relationship.

**Methods:**

Utilizing summary statistics of genome-wide association studies (GWAS), we conducted univariate MR to estimate 2 menstrual factors (Age at menarche, AAM; Age at menopause, AMP) and 5 reproductive factors (Age at first live birth, AFB; Age at last live birth, ALB; Number of live births, NLB; Age first had sexual intercourse, AFSI; Age started oral contraceptive pill, ASOC) on OA (overall OA, OOA; knee OA, KOA and hip OA, HOA). The sample size of MRF ranged from 123846 to 406457, and the OA sample size range from 393873 to 484598. Inverse variance weighted (IVW) method was used as the primary MR analysis methods, and MR Egger, weighted median was performed as supplements. Sensitivity analysis was employed to test for heterogeneity and horizontal pleiotropy. Finally, multivariable MR was utilized to adjust for the influence of BMI on OA.

**Results:**

After conducting multiple tests (*P*<0.0023) and adjusting for BMI, MR analysis indicated that a lower AFB will increase the risk of OOA (odds ratio [OR] = 0.97, 95% confidence interval [CI]: 0.95–0.99, P = 3.39×10^−4^) and KOA (OR = 0.60, 95% CI: 0.47–0.78, *P* = 1.07×10^−4^). ALB (OR = 0.61, 95% CI: 0.45–0.84, *P* = 2.06×10^−3^) and Age AFSI (OR = 0.66, 95% CI: 0.53–0.82, *P* = 2.42×10^−4^) were negatively associated with KOA. In addition, our results showed that earlier AMP adversely affected HOA (OR = 1.12, 95% CI: 1.01–1.23, *P* = 0.033), and earlier ASOC promote the development of OOA (OR = 0.97, 95% CI: 0.95–1.00, *P* = 0.032) and KOA (OR = 0.58, 95% CI: 0.40–0.84, *P* = 4.49×10^−3^). ALB (OR = 0.98, 95% CI: 0.96–1.00, *P* = 0.030) and AFSI (OR = 0.98, 95% CI: 0.97–0.99, *P* = 2.66×10^−3^) also showed a negative association with OOA but they all did not pass multiple tests. The effects of AAM and NLB on OA were insignificant after BMI correction.

**Conclusion:**

This research Certificates that Early AFB promotes the development of OOA, meanwhile early AFB, ALB, and AFSI are also risk factors of KOA. Reproductive factors, especially those related to birth, may have the greatest impact on KOA. It provides guidance for promoting women’s appropriate age fertility and strengthening perinatal care.

## Introduction

Osteoarthritis (OA) is the most common form of degenerative joint disease, affecting approximately 302 million people worldwide [1]. It often leads to long-term chronic pain and impaired joint mobility, significantly impacting the quality of life of those affected [2]. With an aging population and changing lifestyle, the increasing prevalence of OA, primarily due to age and obesity, has emerged as a significant public health concern [3].

The incidence of OA varies between genders. Epidemiologic studies indicate that OA is significantly more prevalent in females than in males, with the World Health Organization reporting incidence rates of 18% in women compared to 9.8% in men [4, 5]. Unique physiological characteristics associated with menstruation and reproduction in women suggest a possible link to OA. Notably, the incidence of OA significantly increases after menopause, indicating that changes in menstrual reproductive factors (MRF) may influence the development of OA [5–8]. One prospective cohort study showed that a later age at menarche (AAM) was related to a lower risk of developing OA, and that past users or users of oral contraceptives or taking hormone replacement therapy increased the risk of joint replacement in OA [9]. *Riyazi’s* study showed that later age at menopause (AMP) decrease the risk of familial OA [10]. However, there are some different views believe that later AAM Increases the likelihood of knee and hip replacement surgery [11], and menopausal status or AMP are not a predictor of developing OA [12]. The Number of live births (NLB) has also been studied, with mixed finding on its impact on OA [9, 13–15].

Despite numerous observational studies demonstrating a potential association between MRF and OA, the precise nature of this association requires further clarification. Observational studies are subject to confounding effects and horizontal pleiotropy, which can obscure the distinction between reverse causality and lead to variable results, thus undermining their overall credibility. Mendelian randomization (MR) offers a more robust approach. This analytical method examines potential causal associations between dangerous factors and outcomes using data from genome-wide association studies (GWAS) [16]. The genetic variants associated with exposure were used as instrumental variables (IVs) to match the outcome, and MR analysis allowed for a more intuitive response to reflect causal relationship [17, 18]. By adhering to the principles of random assignment of Mendelian gametes and free combination, MR effectively mitigates issues of confounding factors and reverse causality [17].

In this study, we used a two-sample MR method to assess the possible causal relationship between MRF and OA, and adjust for the influence of BMI, with a view to providing guidance for the prevention and treatment of female reproductive health and OA.

## Materials and methods

### Study design

**Fig 1:**
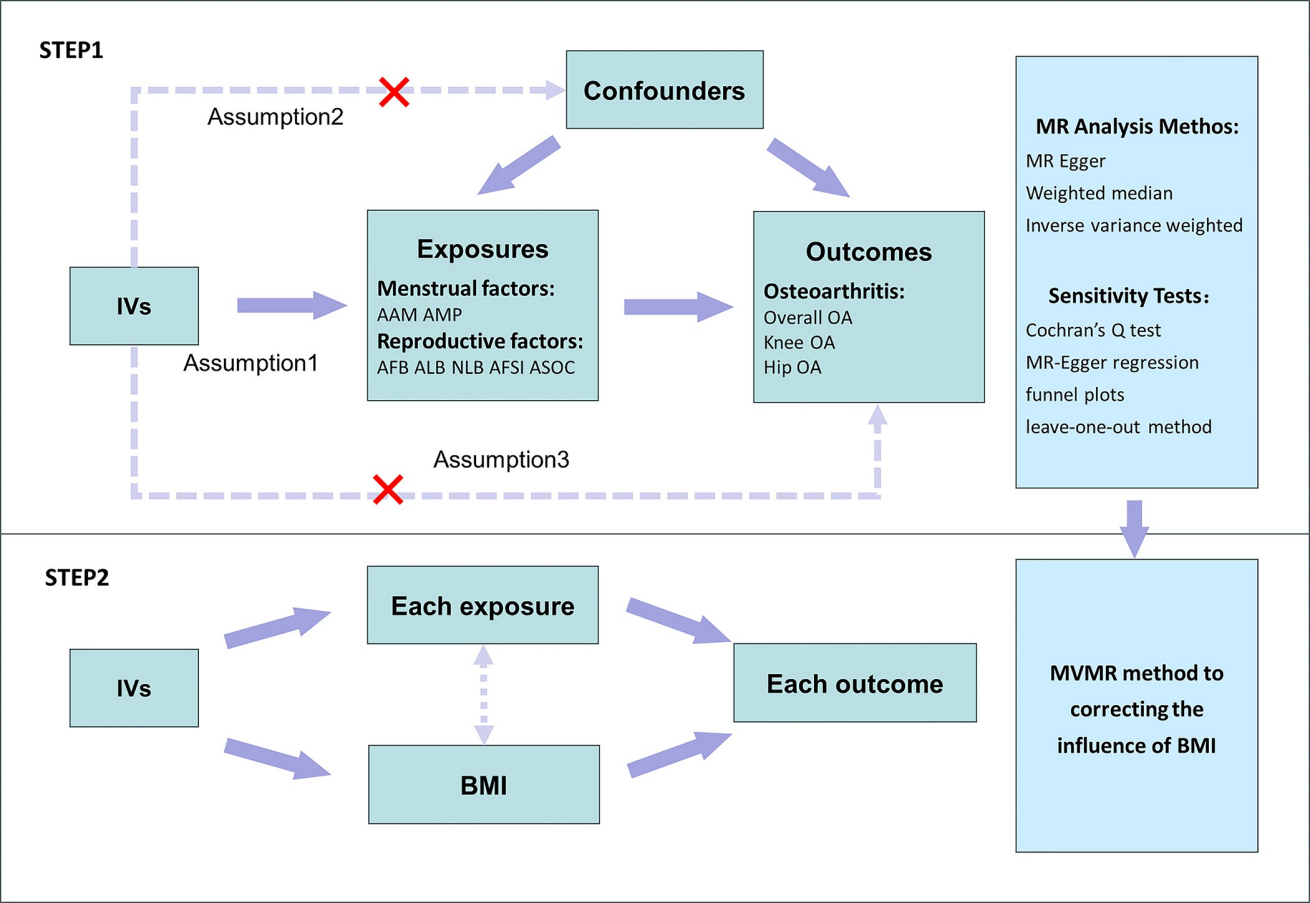
A summary of the study design. IVs, instrumental variables; AAM, Age at menarche; AMP, Age at menopause; AFB, Age at first live birth; ALB, Age at last live birth; NLB, Number of live births; AFSI, Age first had sexual intercourse; ASOC, Age started oral contraceptive pill; OA, osteoarthritis; BMI Body mass index.

This two-sample MR analysis consists of two steps. In the first step, we validate the causal association between seven exposure factors related to menstruation (AAM, AMP) and reproduction (Age at First Birth,AFB; Age at Last Birth, ALB; NLB; Age First Had Sexual Intercourse,AFSI; Age Started Oral Contraceptive Pill, ASOC) and three outcomes (Overall osteoarthritis, OOA; knee osteoarthritis, KOA; and hip osteoarthritis, HOA) using a univariate MR (UNMR) method. In the second step, we adjust the effect of Body Mass Index (BMI) on OA through a multivariate MR (MVMR) method. We use Single nucleotide polymorphisms (SNPs) that are significantly correlated with MRF as instrumental variables (IVs). Additionally, in step one, sensitivity analysis was employed to test the stability of IVs,including Cochran Q test, MR-Egger regression, funnel plot, and the leave-one-out method. This design approach has been referenced in several articles [19, 20]. For SNPs to serve effectively as IVs, they must fulfill the following three assumptions: (1) the relevance assumption, where SNPs are strongly correlated with MRF; (2) the independence assumption, where SNPs are not associated with confounders; and (3) the exclusionary assumption, where SNPs are not straightforwardly involved in the outcome but operate through the way of exposure [21]. This study design is shown in Fig 1.

### Data sources and SNP selection for exposures and outcomes

All the pooled data for this two-sample MR study were sourced from the publicly accessible IEU GWAS database summary website (https://gwas.mrcieu.ac.uk/). The selected exposures and outcomes originated from different datasets to prevent sample overlap. The exposure data were exclusively derived from the UK Biobank database, while the outcome data came from the EBI database. Both databases primarily encompass European populations. A detailed summary of this information is presented in Table 1.

**Table 1:** Details of GWAS data used in the study.

Exposure data	Unit	Sample size	Consortium	Ancestry	GWAS-ID	Year
**AAM**	SD	243944	MRC-IEU	European	ukb-b-3768	2018
**AMP**	SD	143819	MRC-IEU	European	ukb-b-17422	2018
**AFB**	SD	123846	Neale Lab	European	ukb-a-319	2017
**ALB**	SD	170248	MRC-IEU	European	ukb-b-8727	2018
**NLB**	SD	180952	Neale Lab	European	ukb-a-317	2017
**AFSI**	SD	406457	MRC-IEU	European	ukb-b-6591	2018
**ASOC**	SD	198213	MRC-IEU	European	ukb-b-9433	2018
**Outcome data**	Unit	Sample size	Consortium	Ancestry	GWAS-ID	Year
**OOA**	Event	484598	NA	European	ebi-a-GCST90038686	2021
KOA	Event	403124	NA	European	ebi-a-GCST007090	2019
HOA	Event	393873	NA	European	ebi-a-GCST007091	2019
Confounder	Unit	Sample size	Consortium	Ancestry	GWAS-ID	Year
BMI	SD	461460	MRC-IEU	European	ukb-b-19953	2018

AAM, Age at menarche; AMP, Age at menopause; AFB, Age at first live birth; ALB, Age at last live birth; NLB, Number of live births; AFSI, Age first had sexual intercourse; ASOC, Age started oral contraceptive pill; OOA, overall osteoarthritis; KOA, knee osteoarthritis; HOA. hip osteoarthritis; BMI. Body mass index; SD, standard deviation; NA, not applicable.

In order to ensure the selected IVs are representative and minimize bias, we adopted the following criteria for selecting SNPs: We screened for SNPs with a strong significant association (*P*<5.0×10^−8^) with the exposure and removed those in linkage disequilibrium (r^2^ = 0.001, kb = 10000). We used the LDTrait tool (https://ldlink.nih.gov/?tab=ldtrait) to complete the independence and exclusivity assumptions [22]. After the initial screening of IVs for exposures we matched the SNPs information for the outcomes to obtain SNPs of outcomes. And proxy SNPs were not used. SNPs with palindromic structures and minor allele frequency (MAF) greater than 0.01 were eliminate. We calculated the F-statistic to measure whether the selected SNPs were weak instrumental variable. an F<10, indicates the presence of a weakly instrumented variable. The F-value is calculated from the following formula: F = R^2^(N − k − 1)/k(1 − R^2^) [23], where N is the sample size of the exposed database, k is the number of selected SNPs, and R^2^ is the proportion of variance explained by each SNP. R^2^ is further calculated as: R^2^ = [2×Beta^2^ × (1− EAF) × EAF] / [2 × Beta^2^ × (1− EAF) ×EAF+2×SE^2^ × N × (1− EAF) × EAF], where EAF is the effect allele frequency, Beta is the allele effect value, and SE is the standard error.

### MR analysis

After obtaining SNP information for each exposures and outcomes, we harmonized their SNPs information to ensure that alleles were orientated in the same direction, and removed palindromic SNPs and incompatible SNPs. The primary analysis technique employed was the Inverse Variance Weighted (IVW) method. It based on the assumption that SNPs are valid IVs. This method examines the causal association by conducting a meta-analysis of each Wald ratio for the selected SNPs [24, 25]. MR-Egger and Weighted Median (WM) served as complementary methods of IVW method. MR-Egger presumes that each SNP has an uncorrelated association with horizontal pleiotropic effects, which may lead to inaccurate results, especially when the quantity of genetic instruments is limited [26]. The WM method, calculates the causal effect using the median of the estimates and remains consistent even if up to 50% of the SNPs are derived from invalid IVs [27]. The IVW method has the highest test efficacy of all the analyses, so we use the IVW approach as the main method of analysis, and we consider such results to be significant as long as the IVW results are significant and the direction of the β values of the other two methods is the same. The MR-Egger method has a low test efficacy, but its intercept value can be used as an assessment of horizontal multivariate validity. To adjust for BMI effect in our models, we conducted Multivariate MR (MVMR) analysis, which also used the IVW method [28]. It involves selecting all exposures for SNPs and regressing them against the outcome collectively, with weights determined by the inverse variance of the outcome. This method helps limit the indirect pathway effects of exposure-SNP on other risk factors. The TwoSampleMR (version 0.5.7) package in R software (version 4.3.2) was used as the main analytical tool. A *P* value of less than 0.0023 (0.05/21, with Bonferroni correction) was considered significant.

### Sensitivity analysis

After selecting the IVs and deriving the statistical results from the MR analysis, we also need to perform a sensitivity analysis on the results. In this study, Cochran’s Q test, MR-Egger regression analysis, funnel plots, and the leave-one-out method were employed for this purpose. Cochran’s Q statistics were utilized to explore heterogeneity among IVs [29]. If the Cochran’s Q assay is shown to be heterogeneous, we transitioned from a fixed effect to a random effects model, as the latter is more tolerant of heterogeneity. Funnel plots served as a visual tool for observing horizontal pleiotropy, with symmetry in the graphs indicating a lower likelihood of pleiotropy [27]. The MR-Egger method was also employed to assess horizontal pleiotropy. A significant deviation from zero in the intercept term of the MR-Egger regression signals horizontal pleiotropy [30]. Additionally, the leave-one-out method was used to determine whether the statistical results were influenced by a single SNP by sequentially removing each SNP in turn. In these tests all P-value was employed by 0.05 as statistically significant results.

## Results

### Results of instrumental variables selection

After filtering SNPs by setting the P-value less than 5×10^−8^ and removing the interference of linkage disequilibrium, as well as harmonizing the OA information from the GWAS database, we ultimately identified 523 SNPs as IVs (S1 Table). These included 194 for AAM, 109 for AMP, 17 for AFB, 6 for ALB, 3 for NLB, 190 for AFSI and 4 for ASOC. The minimum F-statistic value was 32.16, and the maximum was 95.39 (as detailed in S1 Table). All values were greater than 10, indicating that the IVs could strongly predict OA in this study.

### Results of Univariable MR analysis

**Fig 2:**
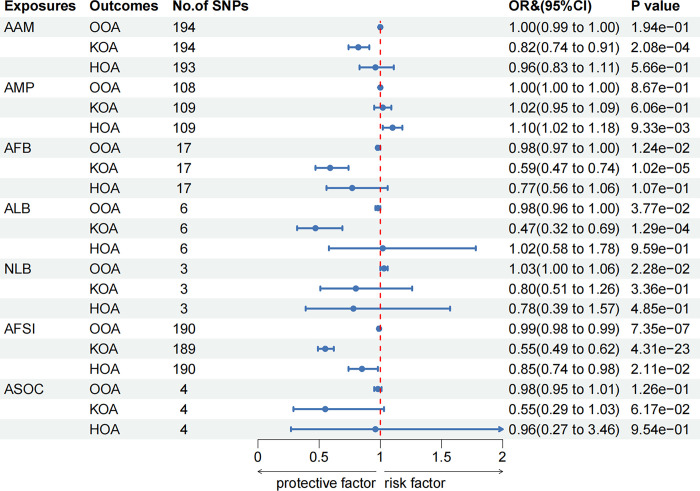
Univariable MR results of MRF on the risk of OA. All presented results are derived from IVW analysis.

After conducting multiple tests (*P*<0.0023), the genetically predicted results are as follows: Among the two menstrual factors, each additional standard deviation (SD) increase in AAM was associated with a decreased risk of KOA [IVW: odds ratio (OR) = 0.82, 95% confidence interval (CI): 0.74–0.91, *P* = 2.08×10^−4^]. However, AMP has not casual association with any form of OA. From the five reproductive factors, as each SD increased, AFB, ALB and AFSI all lowered the risk of OA. Specifically, AFB only had a causal association with KOA (IVW: OR = 0.59, 95% CI: 0.47–0.74, *P* = 1.02×10^−5^), ALB only with KOA (IVW: OR = 0.47, 95% CI: 0.32–0.69, *P* = 1.29×10^−4^), and AFSI with both OOA (IVW: OR = 0.99, 95% CI: 0.98–0.99, *P* = 7.35×10^−7^) and KOA (IVW: OR = 0.55, 95% CI: 0.49–0.62, *P* = 4.31×10^−23^). And, in the above results, the direction of the β values obtained by the MR-Egger and WM methods are the same as the IVW results. Without considering multiple tests, there was also a causal relationship observed between AMP and HOA (IVW: OR = 1.10, 95% CI: 1.02–1.18, *P* = 9.33×10^−3^), AFB and OOA (IVW: OR = 0.98, 95% CI: 0.97–1.00, *P* = 0.012), ALB and OOA (IVW: OR = 0.98, 95% CI: 0.96–1.00, *P* = 0.038), NLB and OOA (IVW: OR = 1.03, 95% CI: 1.00–1.06, *P* = 0.023), and AFSI and HOA (IVW: OR = 0.85, 95% CI: 0.74–0.98, *P* = 0.021), as their p-values fell between 0.0023 and 0.05. The IVW results are depicted in Fig 2, with MR-Egger and WM results provided in S2 Table.

### Results of Multivariable MR correction

**Fig 3:**
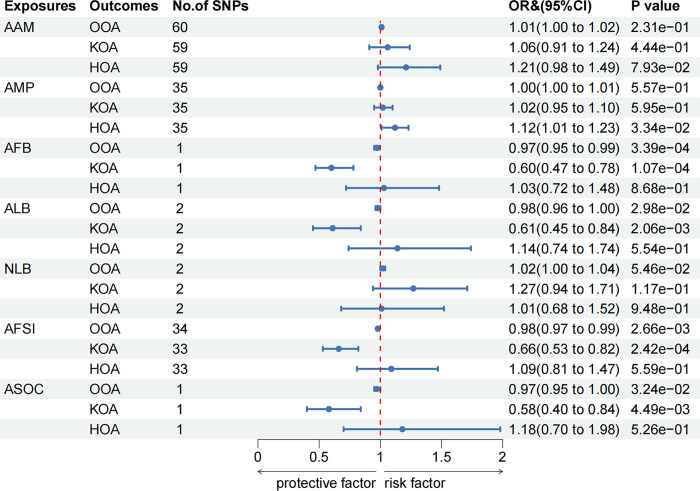
Multivariable MR results of MRF on risk of OA after adjusting BMI.

**Fig 4:**
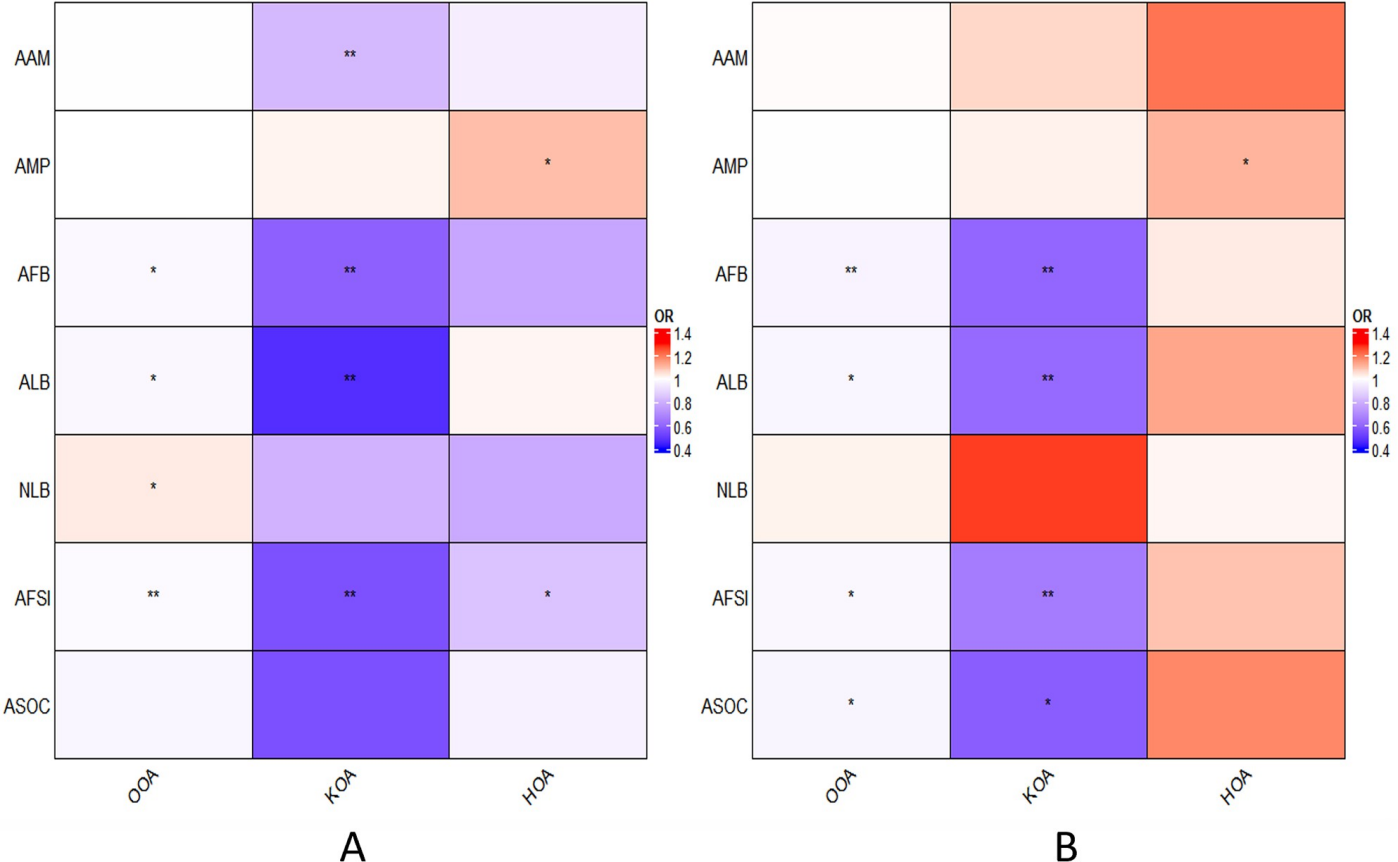
Heatmap of seven exposure factors with three OA outcomes. A. The results of Univariable MR analysis, B. The results of Multivariable MR analysis. **P<0.0023, *0.0023<*P*<0.05, OR odds ratio.

After correcting for BMI, the causal association between AAM and KOA was insignificant (IVW: OR = 1.06, 95% CI: 0.91–1.24, *P* = 0.44). However, negative relationships persisted between AFB (IVW: OR = 0.60, 95% CI: 0.47–0.78, *P* = 1.07×10^−4^), ALB (IVW: OR = 0.61, 95% CI: 0.45–0.84, *P* = 2.06×10^−3^) and AFSI (IVW: OR = 0.66, 95% CI: 0.53–0.82, *P* = 2.42×10^−4^) with KOA. After MVMR correction, the association of AFB with OOA became significant (IVW: OR = 0.97, 95% CI: 0.95–0.99, *P* = 3.39×10^−4^) (Fig 3). Additionally, an earlier AMP was associated with a detrimental effect on HOA (IVW: OR = 1.12, 95% CI: 1.01–1.23, *P* = 0.033), and an earlier ASOC was linked to the development of OOA (IVW: OR = 0.97, 95% CI: 0.95–1.00, *P* = 0.032) and KOA (IVW: OR = 0.58, 95% CI: 0.40–0.84, *P* = 4.49×10^−3^). ALB (IVW: OR = 0.98, 95% CI: 0.96–1.00, *P* = 0.030) and AFSI (IVW: OR = 0.98, 95% CI: 0.97–0.99, *P* = 2.66×10^−3^) also demonstrated negative associations with OOA, but they did not pass multiple tests. The results of the Multivariable MR are depicted in Fig 3, with additional details available in S3 Table. Fig 4 presents a comparison of the results from UVMR and MVMR.

### Result of sensitivity test

The Cochran Q test showed that IVs of AAM, AMP, AFB, AFSI and ASOC have heterogeneity. Consequently, we opted for the random effects model of the IVW method (Table 2). The funnel plot shows that all graphs are symmetric, suggesting no significant outliers (S1 Fig). The MR-Egger results demonstrated that all p-values were bigger than 0.05, demonstrating the effect of instrumental variables away from horizontal polytropy and can adequately represent the exposure factors without interference from confounders (Table 2). The leave-one-out method confirmed that after sequentially rule out each SNP, the estimates remained consistent with the value calculated using all SNPs, proving that the IVs were robust with little influence from individual SNPs (S2 Fig).

**Table 2:** Pleiotropy and heterogeneity tests for the causal association between MRF on OA.

Exposures	Outcomes	Cochran’s Q test		IVW (random)		Horizontal pleiotropy test	
		Method	Q pval	OR (95% CI)	P value	Intercept	P value
**AAM**	**KOA**	IVW	2.53E-10*	0.82 (0.74, 0.91)	2.08E-04	-9.94E-04	0.73
	**HOA**	IVW	2.73E-20*	0.96 (0.83, 1.11)	5.66E-01	-2.72E-03	0.51
	**OA**	IVW	2.02E-03*	1.00 (0.99, 1.00)	1.94E-01	-1.65E-04	0.26
**AMP**	**KOA**	IVW	1.45E-07*	1.02 (0.95, 1.09)	6.06E-01	-2.43E-04	0.94
	**HOA**	IVW	9.90E-03*	1.10 (1.02, 1.18)	9.33E-03	3.48E-03	0.28
	**OA**	IVW	1.02E-03*	1.00 (1.00, 1.00)	8.67E-01	-1.59E-04	0.32
**AFB**	**KOA**	IVW	8.33E-02	NE	NE	7.97E-03	0.66
	**HOA**	IVW	3.33E-02*	0.77 (0.56, 1.06)	1.07E-01	9.99E-04	0.97
	**OA**	IVW	1.24E-01	NE	NE	6.97E-04	0.50
**ALB**	**KOA**	IVW	2.72E-01	NE	NE	4.76E-03	0.81
	**HOA**	IVW	1.35E-01	NE	NE	1.76E-02	0.52
	**OA**	IVW	4.82E-01	NE	NE	-1.01E-03	0.28
**NLB**	**KOA**	IVW	5.21E-01	NE	NE	-3.34E-02	0.57
	**HOA**	IVW	2.18E-01	NE	NE	-9.27E-02	0.33
	**OA**	IVW	3.63E-01	NE	NE	8.88E-04	0.84
**AFSI**	**KOA**	IVW	9.62E-09*	0.55 (0.49, 0.62)	4.31E-23	-2.62E-03	0.57
	**HOA**	IVW	4.20E-06*	0.85 (0.74, 0.98)	2.11E-02	1.53E-03	0.78
	**OA**	IVW	5.09E-02	NE	NE	-2.04E-04	0.36
**ASOC**	**KOA**	IVW	1.96E-01	NE	NE	3.19E-02	0.33
	**HOA**	IVW	7.14E-03*	0.96 (0.27, 3.46)	9.54E-01	-5.97E-02	0.37
	**OA**	IVW	8.01E-01	NE	NE	4.69E-04	0.75

AAM, Age at menarche; AMP, Age at menopause; AFB, Age at first live birth; ALB, Age at last live birth; NLB, Number of live births; AFSI, Age first had sexual intercourse; ASOC, Age started oral contraceptive pill; OOA, overall osteoarthritis; KOA, knee osteoarthritis; HOA. hip osteoarthritis; NE, not estimate; OR, odds ratio.

*P<0.05.

## Discussion

In this study, we employed two-sample MR methods to investigate the causal association between two menstrual factors, five reproductive factors and three types of OA. Recognizing that BMI is the most common confounder of OA, we adjusted for its effects using a multivariate MR method. After conducting multiple tests, three exposure factors were conclusively identified as having a significant causal association with OA: AFB, ALB and AFSI.

Both AFB and ALB are negatively causally associated with KOA, and additionally, AFB is also negatively associated with OOA. Although AFB and ALB represent different aspects of reproductive age, a smaller ALB also implies an earlier age of childbearing for the same number of children, thereby giving both exposure factors equivalent meanings. This suggests that an earlier reproductive age increases the risk of both KOA and OOA. This aligns with the findings of some observational studies. For instance, a study involving 28 non-pregnant female participants found that the parous group exhibited smaller knee and hip moments and greater knee flexion angles compared to the nulliparous group [31]. This altered habitual loading pattern increases irritation of the cartilage, thereby heightening the risk of developing OA. This suggests that during normal delivery, the expulsion of the fetus affects the vaginal and pelvic structures of the mother, which may, in turn, lead to changes in the force lines of the lower extremities. The alteration in the force lines of the lower limbs may elevate the risk of OA [32, 33]. Early childbirth could potentially lead to early knee joint injuries, and a greater number of births may lead to significant changes in the force lines of the lower limbs. Another cohort study of 4.6 million Danish people indicated that the risk of OOA in women increased by an average of 1.05 times for each additional child, with 1.10 times increase in the risk of KOA [15]. A longitudinal observational study of 1,618 OA patients aged 50–79 years demonstrated that the peril of OA increased with the amounts of births, particularly in women who had more than four children [34]. While our results show great similarity with these studies, the NLB was not significant after BMI correction and multiple tests. However, the genetic estimation results of NLB indicated a positive association with OA (OR = 1.03, *P* = 0.023). Regarding the effect of parity on OA, Wei offered an alternate explanation by suggesting that an increase in number of productions is associated with a decrease in the amount of cartilage in various knee compartments and an increase in cartilage defects [35].

Sexual Intercourse has been linked to pregnancy and childbirth, with earlier sexual activity potentially indicating an earlier pregnancy or birth. A study involving 10164 college students show that early sexual behavior promotes the risk of unintended pregnancy, with an astonishing 34.03% of participants experiencing unintended pregnancies [36]. While there are no direct observational studies establishing that early sexual behavior increases the risk of OA, related research has shown that early sexual intercourse can exacerbate depressive behaviors [37]. This is significant as internalized behaviors like adolescent anxiety, depression, and withdrawnness are known to promote early sexual activity [38]. Early sexual debut heightens the risk of teenage pregnancies, births, and miscarriages [39], which indirectly become fertility factors influencing earlier age and an increased number of births. This, in turn, affects the prevalence of OA.

Many experts argue that the decline in postmenopausal sex hormone levels plays a pivotal role in the formation of OA [40, 41]. They suggest that postmenopausal hormone replacement therapy can mitigate the progression of OA [42, 43]. However, earlier use of hormone therapy or oral contraceptives does not necessarily confer protection against OA. Observational studies have reported an elevated risk of total knee replacement among individuals who had previously used oral contraceptives or undergone hormone replacement therapy [9]. Our findings similarly indicate that earlier ASOC use may tend to accelerate the development of both KOA and OOA. This earlier use of hormone therapy or ASOC might reflect an earlier decline in sex hormone levels in the body.

The impact of AAM on KOA ceased to be significant after adjusting for BMI, indicating a strong correlation between earlier AAM and obesity. This relationship has been substantiated by prior studies demonstrating that higher body weight increases the risk of early menarche in women [44, 45]. Owing to the inability of observational studies to fully exclude the influence of confounding factors, the conclusion that earlier AAM promotes the risk of OA, especially when considering the combined effects of BMI and AAM, has been drawn from prior research [9, 13]. Another cross-sectional study supports our hypothesis, confirming that AAM is not associated with the risk of OA after adjusting for confounding factors [14]. This suggests that AAM may not be an independent risk factor for OA but may influence OA development as a consequence of obesity. Numerous studies have indicated that the morbidity of OA significantly increases in women post-menopause [46, 47]. Our study’s results show that an earlier AMP does not elevate the risk of OOA, and although it retains a significant association with HOA after BMI correction, it did not remain significant after multiple testing. Nevertheless, our findings provide insight into predicting the relationship between AMP and the development of HOA.

Although only AFB, ALB and AFSI were significantly correlated with OA after adjusting for BMI effects and multiple testing, there is unity in all menstrual and reproductive factors on what they illustrate. These individual factors are closely interconnected, where one may be a prerequisite for another. For instance, individuals with earlier AFSI tend to have earlier ASOC and AFB, and those with an earlier AAM may also engage in sexual activity sooner. There is a strong correlation among AAM, AFSI, AFB and NLB [48]. Our results suggest that reproductive factors (AFB, ALB, NLB, AFSI and ASOC) tend to impact OOA and KOA, while menstrual factor (AMP) may influence HOA. The exposure factors we studied (except NLB) almost universally displayed a negative correlation with the outcome. This indicates that later sexual intercourse and later age at childbearing are connecting with a lower danger of OA. These findings are instructive in advocating for avoiding early sexual activity and promoting age-appropriate childbearing.

The advantage of our study lies in utilizing a two-sample MR design and correcting for the influence of BMI using the MVMR method. This approach minimizes the effects of reverse causality and confounding. Additionally, the use of publicly available large sample GWAS datasets for the analysis enhances the accuracy of the results to a certain extent. However, we must acknowledge several potential shortcomings. Firstly, the subjects in our study were primarily of European origin, which may not fully represent the global population. Secondly, menstrual and reproductive factors are intricately linked to changes in human sex hormones, and our study did not account for the effects of various sex hormones on these factors. Furthermore, some exposures yielded a relatively small number of SNPs, potentially affecting the precision of the results. Future research could benefit from exploring larger and more diverse databases.

## Conclusions

In conclusion, our study offers some insights into the impact of ARF on the incidence of OA. We found that relatively early AFB, ALB and AFSI are link with a high risk of developing KOA, and later AFB is associated with a decreased risk of OOA. These findings highlight the potential influence of reproductive life events on OA development. However, further studies are necessary to confirm and extend our observations.

## Supporting information

S1 Table The instrumental variables used in MR analysis between menstrual factors and osteoarthritis. (DOCX)

S2 Table Univariable Mendelian randomization estimates results for menstrual reproductive factors on osteoarthritis. (DOCX)

S3 Table Multivariate Mendelian randomization estimates results for menstrual reproductive factors on osteoarthritis. (DOCX)

S1 Fig Funnel plot of SNPs associated with menstrual reproductive factors and osteoarthritis. (DOCX)

S2 Fig Leave-one-out analysis plot of SNPs associated with menstrual reproductive factors and osteoarthritis. (DOCX)

## References

[1] Kolasinski SL, Neogi T, Hochberg MC, Oatis C, Guyatt G, Block J, et al. 2019 American College of Rheumatology/Arthritis Foundation Guideline for the Management of Osteoarthritis of the Hand, Hip, and Knee. Arthritis Care Res (Hoboken). 2020;72(2):149–162. doi: 10.1002/acr.24131 . 31908149 PMC11488261

[2] Fan Y, Li Z, He Y. Exosomes in the Pathogenesis, Progression, and Treatment of Osteoarthritis. Bioengineering (Basel). 2022;9(3):99. doi: 10.3390/bioengineering9030099 . 35324788 PMC8945849

[3] Hunter DJ, Bierma-Zeinstra S. Osteoarthritis. Lancet. 2019;393(10182):1745–1759. doi: 10.1016/S0140-6736(19)30417-9 . 31034380

[4] Wojcieszek A, Kurowska A, Majda A, Liszka H, Gądek A. The Impact of Chronic Pain, Stiffness and Difficulties in Performing Daily Activities on the Quality of Life of Older Patients with Knee Osteoarthritis. Int J Environ Res Public Health. 2022;19(24):16815. doi: 10.3390/ijerph192416815 . 36554695 PMC9779661

[5] de Klerk BM, Schiphof D, Groeneveld FP, Koes BW, van Osch GJ, van Meurs JB, et al. No clear association between female hormonal aspects and osteoarthritis of the hand, hip and knee: a systematic review. Rheumatology (Oxford). 2009;48(9):1160–5. doi: 10.1093/rheumatology/kep194 . 19608726

[6] Phinyomark A, Osis ST, Hettinga BA, Kobsar D, Ferber R. Gender differences in gait kinematics for patients with knee osteoarthritis. BMC Musculoskelet Disord. 2016;17:157. doi: 10.1186/s12891-016-1013-z . 27072641 PMC4830067

[7] Wang S, Wang H, Liu W, Wei B. Identification of Key Genes and Pathways Associated with Sex Differences in Osteoarthritis Based on Bioinformatics Analysis. Biomed Res Int. 2019;2019:3482751. doi: 10.1155/2019/3482751 . 31886203 PMC6925789

[8] Contartese D, Tschon M, De Mattei M, Fini M. Sex Specific Determinants in Osteoarthritis: A Systematic Review of Preclinical Studies. Int J Mol Sci. 2020;21(10):3696. doi: 10.3390/ijms21103696 . 32456298 PMC7279293

[9] Hellevik AI, Nordsletten L, Johnsen MB, Fenstad AM, Furnes O, Storheim K, et al. Age of menarche is associated with knee joint replacement due to primary osteoarthritis (The HUNT Study and the Norwegian Arthroplasty Register). Osteoarthritis Cartilage. 2017;25(10):1654–1662. doi: 10.1016/j.joca.2017.06.010 . 28705605

[10] Riyazi N, Rosendaal FR, Slagboom E, Kroon HM, Breedveld FC, Kloppenburg M. Risk factors in familial osteoarthritis: the GARP sibling study. Osteoarthritis Cartilage. 2008;16(6):654–9. doi: 10.1016/j.joca.2007.10.012 . 18226556

[11] Eun Y, Yoo JE, Han K, Kim D, Lee KN, Lee J, et al. Female reproductive factors and risk of joint replacement arthroplasty of the knee and hip due to osteoarthritis in postmenopausal women: a nationwide cohort study of 1.13 million women. Osteoarthritis Cartilage. 2022;30(1):69–80. doi: 10.1016/j.joca.2021.10.012 . 34774788

[12] Liu B, Balkwill A, Cooper C, Roddam A, Brown A, Beral V. Reproductive history, hormonal factors and the incidence of hip and knee replacement for osteoarthritis in middle-aged women. Ann Rheum Dis. 2009;68(7):1165–70. doi: 10.1136/ard.2008.095653 . 18957480

[13] Leung YY, Talaei M, Ang LW, Yuan JM, Koh WP. Reproductive factors and risk of total knee replacement due to severe knee osteoarthritis in women, the Singapore Chinese Health Study. Osteoarthritis Cartilage. 2019;27(8):1129–1137. doi: 10.1016/j.joca.2019.03.002 . 30902701 PMC6646081

[14] Wang A, Zawadzki N, Hedlin H, LeBlanc E, Budrys N, Van Horn L, et al. Reproductive history and osteoarthritis in the Women’s Health Initiative. Scand J Rheumatol. 2021;50(1):58–67. doi: 10.1080/03009742.2020.1751271 . 32757806 PMC12447291

[15] Jørgensen KT, Pedersen BV, Nielsen NM, Hansen AV, Jacobsen S, Frisch M. Socio-demographic factors, reproductive history and risk of osteoarthritis in a cohort of 4.6 million Danish women and men. Osteoarthritis Cartilage. 2011;19(10):1176–1182. doi: 10.1016/j.joca.2011.07.009 . 21835256

[16] Bowden J, Holmes MV. Meta-analysis and Mendelian randomization: A review. Res Synth Methods. 2019;10(4):486–496. doi: 10.1002/jrsm.1346 . 30861319 PMC6973275

[17] Smith GD, Ebrahim S. ’Mendelian randomization’: can genetic epidemiology contribute to understanding environmental determinants of disease? Int J Epidemiol. 2003;32(1):1–22. doi: 10.1093/ije/dyg070 . 12689998

[18] Lawlor DA, Harbord RM, Sterne JAC, Timpson N, Davey Smith G. Mendelian randomization: using genes as instruments for making causal inferences in epidemiology. Stat Med. 2008;27(8):1133–1163. doi: 10.1002/sim.3034 . 17886233

[19] Deng MG, Liu F, Liang Y, Wang K, Nie JQ, Liu J. Association between frailty and depression: A bidirectional Mendelian randomization study. Sci Adv. 2023;9(38):eadi3902. doi: 10.1126/sciadv.adi3902 . 37729413 PMC10511184

[20] Wang YB, Yang L, Deng YQ, Yan SY, Luo LS, Chen P, et al. Causal relationship between obesity, lifestyle factors and risk of benign prostatic hyperplasia: a univariable and multivariable Mendelian randomization study. J Transl Med. 2022;20(1):495. doi: 10.1186/s12967-022-03722-y . 36309747 PMC9617448

[21] Sekula P, Del Greco M F, Pattaro C, Köttgen A. Mendelian Randomization as an Approach to Assess Causality Using Observational Data. J Am Soc Nephrol. 2016;27(11):3253–3265. doi: 10.1681/ASN.2016010098 . 27486138 PMC5084898

[22] Ni Y, Zhang D, Tang W, Xiang L, Cheng X, Zhang Y, et al. Body mass index, smoking behavior, and depression mediated the effects of schizophrenia on chronic obstructive pulmonary disease: trans-ethnic Mendelian-randomization analysis. Front Psychiatry. 2024;15:1405107. doi: 10.3389/fpsyt.2024.1405107 . 38846919 PMC11155452

[23] Dan YL, Wang P, Cheng Z, Wu Q, Wang XR, Wang DG, et al. Circulating adiponectin levels and systemic lupus erythematosus: a two-sample Mendelian randomization study. Rheumatology (Oxford). 2021;60(2):940–946. doi: 10.1093/rheumatology/keaa506 . 32944772

[24] Burgess S, Small DS, Thompson SG. A review of instrumental variable estimators for Mendelian randomization. Stat Methods Med Res. 2017;26(5):2333–2355. doi: 10.1177/0962280215597579 . 26282889 PMC5642006

[25] Bowden J, Del Greco M F, Minelli C, Davey Smith G, Sheehan N, Thompson J. A framework for the investigation of pleiotropy in two-sample summary data Mendelian randomization. Stat Med. 2017;36(11):1783–1802. doi: 10.1002/sim.7221 . 28114746 PMC5434863

[26] Bowden J, Davey Smith G, Burgess S. Mendelian randomization with invalid Instruments: effect estimation and bias detection through Egger regression. Int J Epidemiol. 2015;44(2):512–25. doi: 10.1093/ije/dyv080 . 26050253 PMC4469799

[27] Bowden J, Davey Smith G, Haycock PC, Burgess S. Consistent Estimation in Mendelian Randomization with Some Invalid Instruments Using a Weighted Median Estimator. Genet Epidemiol. 2016;40(4):304–14. doi: 10.1002/gepi.21965 . 27061298 PMC4849733

[28] Burgess S, Thompson SG. Multivariable Mendelian randomization: the use of pleiotropic genetic variants to estimate causal effects. Am J Epidemiol. 2015;181(4):251–60. doi: 10.1093/aje/kwu283 . 25632051 PMC4325677

[29] Cohen JF, Chalumeau M, Cohen R, Korevaar DA, Khoshnood B, Bossuyt PM. Cochran’s Q test was useful to assess heterogeneity in likelihood ratios in studies of diagnostic accuracy. J Clin Epidemiol. 2015;68(3):299–306. doi: 10.1016/j.jclinepi.2014.09.005 . 25441698

[30] Burgess S, Thompson SG. Interpreting findings from Mendelian randomization using the MR-Egger method. Eur J Epidemiol. 2017;32(5):377–389. doi: 10.1007/s10654-017-0255-x . 28527048 PMC5506233

[31] Stein BP, Boyer KA. Impact of parity on biomechanical risk factors for knee OA initiation. Gait Posture. 2021;84:287–292. doi: 10.1016/j.gaitpost.2020.12.024 . 33418454

[32] Fan C, Niu Y, Wei M, Kong L, Wang F. Study on the correlation between the severity of patellofemoral arthritis and the morphology of the distal femur. BMC Musculoskelet Disord. 2023;24(1):90. doi: 10.1186/s12891-023-06198-z . 36732733 PMC9893554

[33] Lu Y, Zheng ZL, Lv J, Hao RZ, Yang YP, Zhang YZ. Relationships between Morphological Changes of Lower Limbs and Gender During Medial Compartment Knee Osteoarthritis. Orthop Surg. 2019;11(5):835–844. doi: 10.1111/os.12529 . 31663282 PMC6819278

[34] Wise BL, Niu J, Zhang Y, Felson DT, Bradley LA, Segal N, et al. The association of parity with osteoarthritis and knee replacement in the multicenter osteoarthritis study. Osteoarthritis Cartilage. 2013;21(12):1849–54. doi: 10.1016/j.joca.2013.08.025 . 24029601 PMC3855897

[35] Wei S, Venn A, Ding C, Martel-Pelletier J, Pelletier JP, Abram F, et al. The associations between parity, other reproductive factors and cartilage in women aged 50–80 years. Osteoarthritis Cartilage. 2011;19(11):1307–13. doi: 10.1016/j.joca.2011.07.020 . 21872670

[36] Shu C, Fu A, Lu J, Yin M, Chen Y, Qin T, et al. Association between age at first sexual intercourse and knowledge, attitudes and practices regarding reproductive health and unplanned pregnancy: a cross-sectional study. Public Health. 2016;135:104–13. doi: 10.1016/j.puhe.2016.01.021 . 26927825

[37] Vasilenko SA, Kugler KC, Rice CE. Timing of First Sexual Intercourse and Young Adult Health Outcomes. J Adolesc Health. 2016;59(3):291–297. doi: 10.1016/j.jadohealth.2016.04.019 . 27265422 PMC5002249

[38] Skinner SR, Robinson M, Smith MA, Robbins SC, Mattes E, Cannon J, et al. Childhood behavior problems and age at first sexual intercourse: a prospective birth cohort study. Pediatrics. 2015;135(2):255–63. doi: 10.1542/peds.2014-1579 . 25624381

[39] Heywood W, Patrick K, Smith AM, Pitts MK. Associations between early first sexual intercourse and later sexual and reproductive outcomes: a systematic review of population-based data. Arch Sex Behav. 2015;44(3):531–69. doi: 10.1007/s10508-014-0374-3 . 25425161

[40] Wang YXJ. Menopause as a potential cause for higher prevalence of low back pain in women than in age-matched men. J Orthop Translat. 2016;8:1–4. doi: 10.1016/j.jot.2016.05.012 . 30035087 PMC5987020

[41] Schicht M, Ernst J, Nielitz A, Fester L, Tsokos M, Guddat SS, et al. Articular cartilage chondrocytes express aromatase and use enzymes involved in estrogen metabolism. Arthritis Res Ther. 2014;16(2):R93. doi: 10.1186/ar4539 . 24725461 PMC4060203

[42] Jung JH, Bang CH, Song GG, Kim C, Kim JH, Choi SJ. Knee osteoarthritis and menopausal hormone therapy in postmenopausal women: a nationwide cross-sectional study. Menopause. 2018;26(6):598–602. doi: 10.1097/GME.0000000000001280 . 30586007

[43] Prieto-Alhambra D, Javaid MK, Judge A, Maskell J, Cooper C, Arden NK. Hormone replacement therapy and mid-term implant survival following knee or hip arthroplasty for osteoarthritis: a population-based cohort study. Ann Rheum Dis. 2015;74(3):557–63. doi: 10.1136/annrheumdis-2013-204043 . 24451241

[44] Juul F, Chang VW, Brar P, Parekh N. Birth weight, early life weight gain and age at menarche: a systematic review of longitudinal studies. Obes Rev. 2017;18(11):1272–1288. doi: 10.1111/obr.12587 . 28872224

[45] Matsuo LH, Adami F, Pereira LJ, Silva DAS, de Vasconcelos FAG, Longo GZ, et al. Age at menarche and its association with overweight including obesity and socio-economic conditions of Brazilian schoolgirls: A time-trend analysis. Nutr Bull. 2022;47(1):70–81. doi: 10.1111/nbu.12544 . 36045078

[46] Wen ZQ, Lin J, Xie WQ, Shan YH, Zhen GH, Li YS. Insights into the underlying pathogenesis and therapeutic potential of endoplasmic reticulum stress in degenerative musculoskeletal diseases. Mil Med Res. 2023;10(1):54. doi: 10.1186/s40779-023-00485-5 . 37941072 PMC10634069

[47] Abshirini M, Coad J, Wolber FM, von Hurst P, Miller MR, Tian HS, et al. Effect of Greenshell^TM^ mussel on osteoarthritis biomarkers and inflammation in healthy postmenopausal women: a study protocol for a randomized double-blind placebo-controlled trial. Trials. 2021;22(1):498. doi: 10.1186/s13063-021-05473-5 . 34321048 PMC8317363

[48] Sýkorová K, Flegr J. Faster life history strategy manifests itself by lower age at menarche, higher sexual desire, and earlier reproduction in people with worse health. Sci Rep. 2021;11(1):11254. doi: 10.1038/s41598-021-90579-8 . 34045560 PMC8159921

