# Supplementary material for: Causal association of menstrual reproductive factors on the risk of osteoarthritis: A univariate and multivariate Mendelian randomization study
Source: PLoS One. 2024 Aug 30;19(8):e0307958. doi: 10.1371/journal.pone.0307958 (PMC11364240; doi:10.1371/journal.pone.0307958)
Supplement: S1 Table — (DOCX) [file pone.0307958.s001.docx]

**Supplementary Table 1: The instrumental variables used in MR analysis between menstrual reproductive factors and osteoarthritis.**

| SNP | Chr | Position | Effect allele | Other allele | EAF | Exposure (AAM) | | | Outcome (P value) | | |
| --- | --- | --- | --- | --- | --- | --- | --- | --- | --- | --- | --- |
|  |  |  |  |  |  | Beta | SE | P value | Overall OA | Knee OA | Hip OA |
| rs1005579 | 22 | 22300941 | C | T | 0.454564 | -0.0130876 | 0.00208463 | 3.40E-10 | 0.27 | 0.8146 | 0.1542 |
| rs10063744 | 5 | 95649772 | G | C | 0.284018 | 0.0136863 | 0.00229933 | 2.60E-09 | 0.18 | 0.9108 | 0.9761 |
| rs10090153 | 8 | 77784096 | G | A | 0.601944 | 0.0145673 | 0.00211027 | 5.10E-12 | 0.49 | 0.8649 | 0.01 |
| rs1010961 | 3 | 24254750 | A | T | 0.45165 | -0.0122721 | 0.00207704 | 3.50E-09 | * | * | * |
| rs10136330 | 14 | 30514335 | T | C | 0.039756 | -0.0292842 | 0.00530296 | 3.30E-08 | 0.0280001 | 0.2755 | 0.6623 |
| rs10150677 | 14 | 93917392 | G | C | 0.688695 | -0.01471 | 0.00223286 | 4.50E-11 | 0.55 | 0.793601 | 0.524901 |
| rs10203978 | 2 | 142337743 | T | C | 0.486063 | -0.0142466 | 0.00206734 | 5.50E-12 | 0.49 | 0.3964 | 0.4366 |
| rs10208482 | 2 | 199608595 | A | C | 0.353145 | -0.0233933 | 0.00218003 | 7.30E-27 | 0.59 | 0.701699 | 0.9825 |
| rs10226266 | 7 | 32941119 | G | A | 0.588278 | 0.0122202 | 0.00211082 | 7.10E-09 | 0.42 | 0.6029 | 0.6276 |
| rs10810065 | 9 | 13982870 | C | T | 0.258443 | -0.0140758 | 0.00235456 | 2.30E-09 | 0.32 | 0.193 | 0.2215 |
| rs10860153 | 12 | 97541532 | G | A | 0.336726 | -0.0130358 | 0.00219916 | 3.10E-09 | 0.81 | 0.7959 | 0.7413 |
| rs10861880 | 12 | 108611474 | C | T | 0.503131 | -0.0122745 | 0.0020635 | 2.70E-09 | 0.51 | 0.0808202 | 0.2374 |
| rs10895141 | 11 | 101436741 | C | A | 0.669791 | 0.0206238 | 0.00219864 | 6.60E-21 | 0.13 | 0.8459 | 0.8841 |
| rs11030104 | 11 | 27684517 | G | A | 0.202637 | 0.0196928 | 0.00256689 | 1.70E-14 | 0.0179999 | 0.4922 | 0.8206 |
| rs11031006 | 11 | 30226528 | A | G | 0.142341 | 0.0209309 | 0.00296442 | 1.70E-12 | 0.43 | 0.5066 | 0.32 |
| rs11039266 | 11 | 47532395 | G | T | 0.279482 | 0.0187509 | 0.00229868 | 3.40E-16 | 0.46 | 0.337 | 0.3143 |
| rs11047473 | 12 | 24634741 | A | G | 0.335663 | 0.0140596 | 0.00219213 | 1.40E-10 | 0.450001 | 0.9361 | 0.0224202 |
| rs11130329 | 3 | 52896855 | A | C | 0.858092 | -0.0201635 | 0.00295881 | 9.40E-12 | 0.27 | 0.8222 | 0.9171 |
| rs111401540 | 17 | 6040233 | A | T | 0.255125 | -0.0149353 | 0.00237614 | 3.30E-10 | 0.84 | 0.4986 | 0.2176 |
| rs11218864 | 11 | 122726166 | C | T | 0.141223 | 0.017921 | 0.00296359 | 1.50E-09 | 0.4 | 0.03422 | 0.2053 |
| rs11228700 | 11 | 56419115 | T | C | 0.389066 | -0.0129536 | 0.00212409 | 1.10E-09 | 0.29 | 0.546799 | 0.6457 |
| rs113467400 | 2 | 11720299 | T | C | 0.088509 | -0.0201047 | 0.00365533 | 3.80E-08 | 0.52 | 0.6568 | 0.8908 |
| rs113772882 | 7 | 1642196 | T | G | 0.303595 | -0.012824 | 0.00227968 | 1.90E-08 | 0.41 | 0.1616 | 0.5414 |
| rs114285994 | 16 | 19935763 | A | G | 0.138747 | 0.0247828 | 0.00299305 | 1.20E-16 | 0.0949992 | 0.00166299 | 0.2429 |
| rs115260227 | 4 | 104774698 | G | A | 0.011828 | 0.109998 | 0.00998077 | 3.00E-28 | 0.630001 | 0.2767 | 0.00396798 |
| rs11605776 | 11 | 13318524 | A | C | 0.711371 | -0.0233182 | 0.00228072 | 1.50E-24 | 0.032 | 0.00983309 | 0.4489 |
| rs11619721 | 13 | 112082513 | T | G | 0.091193 | -0.0216554 | 0.00363139 | 2.50E-09 | 0.97 | 0.089869 | 0.815 |
| rs116250833 | 3 | 1855579 | G | A | 0.134521 | 0.0167859 | 0.00305135 | 3.80E-08 | 0.780001 | 0.6023 | 0.3553 |
| rs11678561 | 2 | 164539038 | G | C | 0.268381 | -0.0131328 | 0.00232604 | 1.60E-08 | 0.649999 | 0.6248 | 0.0809897 |
| rs1172955 | 10 | 97877320 | A | T | 0.709964 | -0.0251205 | 0.00227395 | 2.30E-28 | 0.26 | 0.769899 | 0.0358096 |
| rs11738611 | 5 | 156772974 | A | C | 0.213119 | 0.0145574 | 0.00251762 | 7.40E-09 | 0.719999 | 0.3654 | 0.0319499 |
| rs11746435 | 5 | 137006762 | T | A | 0.232121 | 0.0142859 | 0.00245223 | 5.70E-09 | 0.51 | 0.8774 | 0.6264 |
| rs11875135 | 18 | 57892769 | A | T | 0.070522 | 0.0238611 | 0.00405621 | 4.00E-09 | 0.61 | 0.2398 | 0.0335598 |
| rs11893331 | 2 | 56577406 | G | A | 0.169819 | 0.0331922 | 0.00274896 | 1.40E-33 | 0.0690001 | 0.8983 | 0.4211 |
| rs12040029 | 1 | 213451958 | T | C | 0.122633 | -0.0195567 | 0.00329083 | 2.80E-09 | 0.88 | 0.0849005 | 0.2311 |
| rs12134472 | 1 | 199865112 | T | C | 0.430645 | -0.0157792 | 0.00208275 | 3.60E-14 | 0.0920005 | 0.771699 | 0.5061 |
| rs12141660 | 1 | 73195560 | T | C | 0.285995 | -0.0130223 | 0.00228325 | 1.20E-08 | 0.53 | 0.4757 | 0.703301 |
| rs12211649 | 6 | 100172277 | A | G | 0.085437 | 0.0350757 | 0.00370445 | 2.80E-21 | 0.9 | 0.9881 | 0.4602 |
| rs12344679 | 9 | 92245439 | A | G | 0.387641 | -0.0120482 | 0.00211922 | 1.30E-08 | 0.67 | 0.9186 | 0.0293299 |
| rs12360772 | 11 | 1899962 | A | G | 0.186596 | -0.0158553 | 0.00266355 | 2.60E-09 | 0.760001 | 0.391 | 0.9794 |
| rs12460629 | 19 | 18836741 | A | G | 0.188297 | 0.0168159 | 0.0026586 | 2.50E-10 | 0.23 | 0.0190099 | 0.9454 |
| rs12532356 | 7 | 121979635 | A | G | 0.173812 | 0.0179869 | 0.00272778 | 4.30E-11 | 0.0219999 | 0.00580297 | 0.0363999 |
| rs12543893 | 8 | 54029169 | T | C | 0.223275 | 0.0173601 | 0.0024823 | 2.70E-12 | 0.3 | 0.8321 | 0.0769892 |
| rs12550650 | 8 | 4550990 | T | C | 0.452215 | 0.0173466 | 0.00208073 | 7.60E-17 | 0.015 | 0.0471998 | 0.4017 |
| rs12592315 | 15 | 68040266 | G | A | 0.545428 | -0.0117319 | 0.0020824 | 1.80E-08 | 0.68 | 0.8273 | 0.843 |
| rs12607903 | 18 | 3817134 | T | C | 0.720833 | -0.0213151 | 0.00231029 | 2.80E-20 | 0.64 | 0.2268 | 0.0276401 |
| rs12641981 | 4 | 45179883 | T | C | 0.433566 | -0.0162869 | 0.00208522 | 5.70E-15 | 0.38 | 0.1448 | 0.4739 |
| rs12728183 | 1 | 102510732 | T | C | 0.534976 | -0.0159044 | 0.00207616 | 1.90E-14 | 0.11 | 0.3733 | 0.4405 |
| rs12878738 | 14 | 60935281 | T | C | 0.695861 | -0.0197574 | 0.0022445 | 1.30E-18 | 0.74 | 0.3011 | 0.0336899 |
| rs12891481 | 14 | 78426863 | T | C | 0.484602 | -0.013301 | 0.00208366 | 1.70E-10 | 0.31 | 0.0674699 | 0.03125 |
| rs13022337 | 2 | 632609 | G | A | 0.828335 | -0.0312604 | 0.00273174 | 2.50E-30 | 0.14 | 0.0625 | 0.0618002 |
| rs13233916 | 7 | 138874416 | G | C | 0.0892 | 0.0237794 | 0.00362132 | 5.20E-11 | 0.56 | 0.0324004 | 0.0107399 |
| rs13279589 | 8 | 87477723 | T | C | 0.244736 | -0.0138306 | 0.00240301 | 8.60E-09 | 0.21 | 0.468 | 0.3461 |
| rs13322435 | 3 | 156795468 | G | A | 0.40442 | -0.016296 | 0.00211422 | 1.30E-14 | 0.450001 | 0.793299 | 0.1608 |
| rs1361108 | 6 | 126767600 | T | C | 0.457596 | -0.0208891 | 0.00207064 | 6.20E-24 | 0.19 | 0.00228102 | 0.2505 |
| rs1362376 | 16 | 52291939 | C | T | 0.191108 | -0.0192016 | 0.00262637 | 2.70E-13 | 0.8 | 0.3022 | 0.278 |
| rs141344917 | 5 | 111134797 | A | G | 0.063042 | -0.0338561 | 0.00433624 | 5.80E-15 | 0.64 | 0.4734 | 0.8418 |
| rs141829833 | 3 | 51776104 | T | C | 0.02286 | 0.0582767 | 0.00701963 | 1.00E-16 | 0.52 | 0.5427 | 0.677901 |
| rs142058842 | 2 | 156621725 | G | C | 0.165985 | 0.0331631 | 0.00276901 | 4.70E-33 | 0.91 | 0.8322 | 0.5183 |
| rs1516883 | 9 | 108907267 | A | G | 0.314844 | -0.0443187 | 0.00222417 | 2.40E-88 | 0.450001 | 0.652299 | 0.00693298 |
| rs1544543 | 10 | 117615483 | G | A | 0.212136 | 0.0165907 | 0.00253673 | 6.10E-11 | 0.0710003 | 0.1761 | 0.2491 |
| rs1555406 | 14 | 101204235 | T | C | 0.117349 | -0.0194845 | 0.00321801 | 1.40E-09 | 0.11 | 0.720701 | 0.1118 |
| rs16841741 | 2 | 209637598 | G | A | 0.109869 | 0.0247088 | 0.00329443 | 6.40E-14 | 0.77 | 0.4169 | 0.512201 |
| rs16860342 | 3 | 185652947 | T | C | 0.425249 | 0.018305 | 0.0020872 | 1.80E-18 | 0.53 | 0.115 | 0.3453 |
| rs17006627 | 2 | 61389609 | C | G | 0.198752 | 0.0200249 | 0.00258974 | 1.10E-14 | 0.94 | 0.3005 | 0.0091601 |
| rs17053711 | 8 | 25311269 | A | G | 0.261685 | 0.0137707 | 0.00234844 | 4.50E-09 | 0.84 | 0.4413 | 0.0963696 |
| rs17817628 | 17 | 53174727 | G | A | 0.288936 | -0.017391 | 0.002285 | 2.70E-14 | 0.75 | 0.1362 | 0.1467 |
| rs183993 | 4 | 95130025 | A | G | 0.586381 | 0.0119606 | 0.00210114 | 1.30E-08 | 0.0290001 | 0.627401 | 0.1229 |
| rs190286519 | 2 | 203365009 | T | A | 0.121026 | -0.0213495 | 0.00315862 | 1.40E-11 | 0.00599998 | 0.5318 | 0.9294 |
| rs192558 | 1 | 165408986 | T | C | 0.36358 | -0.0275333 | 0.00215916 | 3.00E-37 | 0.5 | 0.1634 | 0.4784 |
| rs1933801 | 6 | 105365725 | T | C | 0.679645 | -0.0620524 | 0.00221003 | 1.80E-173 | 0.92 | 0.9749 | 0.01422 |
| rs1981405 | 11 | 77976208 | T | C | 0.120984 | 0.0210751 | 0.00318393 | 3.60E-11 | 0.64 | 0.01563 | 0.093319 |
| rs2046548 | 9 | 11807272 | T | C | 0.221118 | -0.0173582 | 0.00249021 | 3.20E-12 | 0.85 | 0.7443 | 0.6656 |
| rs2048113 | 6 | 54577218 | C | A | 0.314653 | -0.0163414 | 0.0022257 | 2.10E-13 | 0.719999 | 0.2452 | 0.9338 |
| rs2049618 | 12 | 19988923 | T | A | 0.278544 | -0.0127591 | 0.00231041 | 3.30E-08 | 0.760001 | 0.2494 | 0.9565 |
| rs2051189 | 21 | 35322015 | C | A | 0.50761 | -0.0124474 | 0.00207024 | 1.80E-09 | 0.4 | 0.8262 | 0.01585 |
| rs2067711 | 9 | 105706469 | G | T | 0.277622 | -0.012672 | 0.00230361 | 3.80E-08 | 0.57 | 0.784599 | 0.000462402 |
| rs2076308 | 6 | 50791640 | C | G | 0.180245 | -0.0186496 | 0.00268246 | 3.60E-12 | 0.38 | 0.1025 | 0.1289 |
| rs2123990 | 11 | 8400506 | G | C | 0.413939 | 0.0181483 | 0.00210395 | 6.40E-18 | * | 0.5104 | 0.736999 |
| rs2167378 | 2 | 200045258 | T | C | 0.43765 | -0.0183531 | 0.00207609 | 9.60E-19 | 0.0129999 | 0.1047 | 0.721 |
| rs2234362 | 19 | 36203335 | C | G | 0.05511 | 0.0333845 | 0.00453332 | 1.80E-13 | 0.450001 | 0.3871 | 0.3323 |
| rs2254479 | 6 | 41831143 | A | G | 0.550438 | -0.015924 | 0.0020692 | 1.40E-14 | 0.0690001 | 0.7509 | 0.692299 |
| rs2297605 | 9 | 127255448 | A | G | 0.46171 | 0.0184958 | 0.00209753 | 1.20E-18 | 0.92 | 0.0901509 | 0.8949 |
| rs2343506 | 1 | 162892924 | C | A | 0.598116 | 0.0133464 | 0.00211915 | 3.00E-10 | 0.49 | 0.4587 | 0.7949 |
| rs2422137 | 1 | 72619961 | A | C | 0.453986 | -0.0143644 | 0.00207399 | 4.30E-12 | 0.34 | 0.3738 | 0.4487 |
| rs2428493 | 6 | 31329135 | A | C | 0.410754 | 0.0170355 | 0.00209641 | 4.40E-16 | 0.47 | 0.2865 | 0.34 |
| rs2454055 | 10 | 126859832 | C | T | 0.500256 | 0.0156285 | 0.00206421 | 3.70E-14 | 0.61 | 0.4901 | 0.4236 |
| rs246180 | 16 | 14391923 | A | C | 0.325606 | 0.024272 | 0.00222048 | 8.20E-28 | 0.43 | 0.3788 | 2.90E-05 |
| rs2524158 | 6 | 31260318 | C | A | 0.216713 | 0.0191556 | 0.00275415 | 3.50E-12 | 0.22 | 0.005426 | 0.0421396 |
| rs2524158 | 6 | 31260318 | C | A | 0.216713 | 0.0191556 | 0.00275415 | 3.50E-12 | * | * | * |
| rs2613766 | 19 | 5066995 | C | T | 0.473411 | 0.0115009 | 0.00206963 | 2.70E-08 | 0.0369999 | 0.9915 | 0.0128801 |
| rs2659005 | 17 | 79218714 | T | C | 0.443046 | -0.0133095 | 0.00208494 | 1.70E-10 | 0.35 | 0.1514 | # |
| rs2668767 | 18 | 44785302 | C | T | 0.570724 | 0.0247595 | 0.00209109 | 2.40E-32 | 0.7 | 0.555801 | 0.3767 |
| rs2679894 | 2 | 105870779 | G | A | 0.575649 | -0.022806 | 0.00209484 | 1.30E-27 | 0.84 | 0.3163 | 0.6758 |
| rs2697961 | 1 | 14153333 | T | C | 0.841992 | -0.0237364 | 0.00283862 | 6.20E-17 | 0.44 | 0.764599 | 0.7877 |
| rs27161 | 5 | 34928716 | A | C | 0.274026 | -0.0130451 | 0.00232253 | 1.90E-08 | 0.62 | 0.516699 | 0.106 |
| rs2764261 | 6 | 108927842 | G | A | 0.625538 | -0.0153798 | 0.00213257 | 5.50E-13 | 0.55 | 0.0115401 | 0.1308 |
| rs2798224 | 4 | 3267668 | A | G | 0.568396 | 0.0131267 | 0.00208416 | 3.00E-10 | 0.95 | 0.2579 | 0.8486 |
| rs2953330 | 1 | 244024121 | T | G | 0.625411 | 0.0121035 | 0.00213906 | 1.50E-08 | 0.630001 | 0.8344 | 0.8067 |
| rs3136247 | 2 | 48013099 | G | A | 0.192621 | 0.016269 | 0.0026163 | 5.00E-10 | 0.39 | 0.714399 | 0.00421396 |
| rs34768269 | 17 | 77952751 | C | T | 0.191838 | -0.0159629 | 0.00262693 | 1.20E-09 | 0.34 | 0.5836 | 0.9034 |
| rs34774200 | 20 | 54876504 | T | C | 0.206741 | -0.0143834 | 0.00258049 | 2.50E-08 | 0.96 | 0.515 | 0.7026 |
| rs35005436 | 7 | 74134911 | C | T | 0.159281 | -0.0240794 | 0.00283316 | 1.90E-17 | 0.0269998 | 0.566899 | 0.7828 |
| rs35255016 | 20 | 37294739 | T | C | 0.23743 | 0.0152962 | 0.00243101 | 3.10E-10 | 0.26 | 0.3065 | 0.475 |
| rs35375873 | 5 | 43190647 | C | G | 0.111876 | 0.0218534 | 0.00330938 | 4.00E-11 | 0.61 | 0.00633797 | 0.1742 |
| rs35436838 | 9 | 77273910 | G | T | 0.047044 | 0.0376738 | 0.00491902 | 1.90E-14 | 0.92 | 0.3337 | 0.9913 |
| rs35605425 | 6 | 56860021 | T | A | 0.170469 | -0.0233603 | 0.0027463 | 1.80E-17 | 0.709999 | 0.726901 | 0.8504 |
| rs35917007 | 19 | 1849147 | G | A | 0.533541 | 0.0194369 | 0.00206747 | 5.40E-21 | 0.780001 | 0.1006 | 0.1934 |
| rs35961679 | 17 | 77832212 | A | G | 0.255262 | -0.0130655 | 0.00237415 | 3.70E-08 | 0.15 | 0.48 | 0.782 |
| rs36087932 | 5 | 135615616 | A | G | 0.137749 | 0.0170261 | 0.0029996 | 1.40E-08 | 0.61 | 0.4419 | 0.0470295 |
| rs3743266 | 15 | 60781513 | C | T | 0.335744 | -0.0201453 | 0.00219324 | 4.10E-20 | 0.760001 | 0.1468 | 0.501 |
| rs3810291 | 19 | 47569003 | A | G | 0.675861 | -0.0170572 | 0.00220664 | 1.10E-14 | 0.97 | 0.02426 | 0.2682 |
| rs3827103 | 20 | 54824029 | A | G | 0.08291 | 0.0224695 | 0.00374531 | 2.00E-09 | 0.13 | 0.6271 | 0.339 |
| rs3828002 | 20 | 19702049 | A | G | 0.548329 | 0.0129467 | 0.00209417 | 6.30E-10 | 0.23 | 0.7879 | 0.6182 |
| rs4073513 | 11 | 46071253 | G | A | 0.709481 | 0.0137912 | 0.00226698 | 1.20E-09 | 0.19 | 0.0231201 | 0.4955 |
| rs4141153 | 7 | 41467946 | G | A | 0.196936 | 0.0281279 | 0.00259589 | 2.30E-27 | 0.57 | 0.0452095 | 0.498101 |
| rs4326331 | 7 | 157951887 | C | T | 0.66807 | -0.0124925 | 0.00218955 | 1.20E-08 | 0.719999 | 0.8691 | 0.3566 |
| rs4515148 | 4 | 28789879 | T | G | 0.729071 | 0.0155879 | 0.00232402 | 2.00E-11 | 0.55 | 0.9548 | 0.2163 |
| rs4660257 | 1 | 44148168 | C | T | 0.298682 | -0.0202852 | 0.00225493 | 2.30E-19 | 0.77 | 0.9347 | 0.0500495 |
| rs4664605 | 2 | 153573528 | T | C | 0.647364 | -0.0165058 | 0.00217205 | 3.00E-14 | 0.16 | 0.2953 | 0.0194402 |
| rs4735761 | 8 | 78097161 | C | A | 0.285612 | -0.0181669 | 0.00228449 | 1.80E-15 | 0.87 | 0.00623606 | 0.0349502 |
| rs4738266 | 8 | 73455936 | T | C | 0.682103 | 0.0134075 | 0.00222385 | 1.70E-09 | 0.73 | 0.7949 | 0.2191 |
| rs4755720 | 11 | 43628749 | T | C | 0.607694 | 0.017728 | 0.00212237 | 6.70E-17 | 0.69 | 0.1908 | 0.8851 |
| rs4771121 | 13 | 28018370 | A | G | 0.275093 | 0.0143963 | 0.00232491 | 5.90E-10 | 0.18 | 0.8556 | 0.4166 |
| rs4779051 | 15 | 83370171 | C | A | 0.737649 | -0.0143983 | 0.00235241 | 9.30E-10 | 0.99 | 0.5189 | 0.5354 |
| rs4818008 | 21 | 40611442 | A | T | 0.354674 | -0.0203606 | 0.00216294 | 4.80E-21 | 0.12 | 0.01017 | 0.0139499 |
| rs4857841 | 3 | 128046643 | A | G | 0.267883 | 0.0176633 | 0.00232947 | 3.40E-14 | 0.84 | 0.4273 | 0.3926 |
| rs4875259 | 8 | 3766430 | C | T | 0.6366 | -0.0137417 | 0.0021523 | 1.70E-10 | 0.48 | 0.994 | 0.01911 |
| rs4877593 | 9 | 83221000 | T | A | 0.322914 | -0.0133465 | 0.00220618 | 1.50E-09 | 0.86 | 0.8355 | 0.778599 |
| rs539515 | 1 | 177889025 | C | A | 0.204743 | -0.0288923 | 0.002557 | 1.30E-29 | 0.32 | 0.00527303 | 0.01265 |
| rs545075 | 11 | 94071678 | C | G | 0.94641 | 0.0280331 | 0.00457888 | 9.20E-10 | 0.015 | 0.00846194 | 0.0159199 |
| rs556493 | 6 | 147549297 | G | A | 0.45152 | -0.0127287 | 0.00207619 | 8.70E-10 | 0.73 | 0.6391 | 0.0599294 |
| rs55732507 | 16 | 30141985 | C | T | 0.398766 | 0.0136211 | 0.0021147 | 1.20E-10 | 0.68 | 9.06E-05 | 0.00198198 |
| rs55749651 | 6 | 76294593 | A | G | 0.378749 | 0.0133477 | 0.00212815 | 3.60E-10 | 0.00219999 | 0.8552 | 0.4422 |
| rs55876427 | 13 | 100564082 | A | G | 0.420021 | 0.0140315 | 0.0021084 | 2.80E-11 | 0.27 | 0.02334 | 0.1448 |
| rs561821 | 15 | 41427864 | T | C | 0.37766 | -0.0175362 | 0.00213535 | 2.20E-16 | 0.85 | # | 0.6627 |
| rs5742915 | 15 | 74336633 | C | T | 0.461101 | 0.0161305 | 0.00207305 | 7.20E-15 | 0.17 | 0.4848 | 0.519501 |
| rs583887 | 11 | 65644027 | C | T | 0.801361 | 0.0143892 | 0.00258252 | 2.50E-08 | 0.47 | 0.2882 | 0.6851 |
| rs59072247 | 2 | 73536689 | T | C | 0.030027 | 0.0489499 | 0.00608608 | 8.80E-16 | 0.79 | 0.450199 | 0.6363 |
| rs59695806 | 17 | 43156023 | C | T | 0.29298 | -0.0145498 | 0.00227144 | 1.50E-10 | 1.80E-05 | 0.0229398 | 0.2024 |
| rs6007594 | 22 | 45728370 | A | G | 0.260408 | 0.0128885 | 0.00234641 | 4.00E-08 | 0.14 | 0.00212701 | 0.000358501 |
| rs61371450 | 11 | 16786905 | A | G | 0.176497 | -0.0204651 | 0.00271386 | 4.70E-14 | 0.62 | 0.8504 | 0.9001 |
| rs61655108 | 20 | 33455008 | A | G | 0.239524 | 0.0148592 | 0.00242293 | 8.60E-10 | 1 | 0.3468 | 0.6207 |
| rs61779781 | 1 | 65934354 | C | T | 0.427133 | 0.0149309 | 0.00208358 | 7.70E-13 | 0.5 | 0.0218801 | 0.351 |
| rs62023121 | 15 | 93459453 | T | C | 0.153888 | -0.0177084 | 0.00287298 | 7.10E-10 | 0.2 | 0.9086 | 0.02969 |
| rs62048402 | 16 | 53803223 | A | G | 0.401508 | -0.030691 | 0.0021076 | 4.90E-48 | 0.0899995 | 5.98E-06 | 1.95E-06 |
| rs62106258 | 2 | 417167 | C | T | 0.048774 | 0.0565703 | 0.004783 | 2.80E-32 | 0.12 | 0.000433701 | 0.00158201 |
| rs62306664 | 4 | 132799133 | A | C | 0.19994 | 0.0160662 | 0.00262372 | 9.20E-10 | 0.35 | 0.6187 | 0.8426 |
| rs62376854 | 5 | 110569130 | G | A | 0.057593 | -0.028516 | 0.00448421 | 2.00E-10 | 0.95 | 0.5098 | 0.7795 |
| rs62379978 | 5 | 133915969 | G | T | 0.151825 | 0.0318755 | 0.00290508 | 5.20E-28 | 0.34 | 0.0130599 | 0.1376 |
| rs62507083 | 8 | 53842780 | T | G | 0.119679 | -0.0174403 | 0.00318333 | 4.30E-08 | 0.56 | 0.2134 | 0.649201 |
| rs62520182 | 8 | 140646051 | T | C | 0.148406 | 0.0270222 | 0.00296737 | 8.50E-20 | 0.39 | 0.4907 | 0.4124 |
| rs6499244 | 16 | 69735271 | A | T | 0.560234 | -0.0243808 | 0.00208546 | 1.40E-31 | * | * | * |
| rs6712986 | 2 | 25126046 | C | T | 0.1222 | -0.0211673 | 0.00314581 | 1.70E-11 | 0.44 | 0.0949096 | 0.8081 |
| rs697049 | 9 | 1616908 | T | C | 0.659166 | -0.0153941 | 0.00218158 | 1.70E-12 | 0.16 | 0.4512 | 0.9193 |
| rs7048426 | 9 | 11739159 | A | C | 0.269771 | -0.0159243 | 0.00238254 | 2.30E-11 | 0.99 | 0.4 | 0.1009 |
| rs7048426 | 9 | 11739159 | A | C | 0.269771 | -0.0159243 | 0.00238254 | 2.30E-11 | * | * | * |
| rs7073746 | 10 | 64904071 | G | A | 0.472298 | 0.0134021 | 0.0020697 | 9.50E-11 | 0.0779992 | 0.7502 | 0.0119801 |
| rs7073961 | 10 | 123663470 | T | C | 0.920464 | -0.0215373 | 0.003817 | 1.70E-08 | 0.54 | 0.2438 | 0.0336001 |
| rs7115813 | 11 | 122832074 | G | A | 0.551621 | 0.0280242 | 0.00207902 | 2.10E-41 | 0.2 | 0.0577803 | 0.00457699 |
| rs7132908 | 12 | 50263148 | A | G | 0.384211 | -0.0143563 | 0.00212593 | 1.40E-11 | 0.66 | 0.2983 | 2.72E-05 |
| rs7161194 | 14 | 101529005 | G | A | 0.664093 | 0.0160838 | 0.00228807 | 2.10E-12 | 0.14 | 0.3246 | 0.0120901 |
| rs7178532 | 15 | 23794517 | A | G | 0.695152 | 0.0215465 | 0.00224504 | 8.20E-22 | 0.27 | 0.3385 | 0.721 |
| rs72767997 | 15 | 99288741 | C | T | 0.028534 | 0.0358513 | 0.00630666 | 1.30E-08 | 0.67 | 0.3776 | 0.5359 |
| rs73204208 | 21 | 37791466 | G | T | 0.12898 | 0.0200946 | 0.00309772 | 8.80E-11 | 0.780001 | 0.214 | 0.6505 |
| rs73205548 | 3 | 132607325 | T | C | 0.156374 | 0.0175946 | 0.00285867 | 7.50E-10 | 0.73 | 0.4422 | 0.8599 |
| rs7463166 | 8 | 4833790 | G | A | 0.37395 | -0.0146095 | 0.00213284 | 7.40E-12 | 0.8 | 0.0803397 | 0.05098 |
| rs752278 | 15 | 89037134 | A | G | 0.421367 | -0.0211905 | 0.00212005 | 1.60E-23 | 0.87 | 0.8469 | 0.4063 |
| rs7553348 | 1 | 75005067 | A | G | 0.562415 | 0.0260579 | 0.00208128 | 5.80E-36 | 0.0710003 | 0.1759 | 0.00166901 |
| rs75544266 | 4 | 104584997 | T | C | 0.055947 | 0.0356147 | 0.00449666 | 2.40E-15 | 0.7 | 0.2002 | 0.762 |
| rs7560791 | 2 | 157582917 | C | G | 0.324572 | 0.0121194 | 0.00220734 | 4.00E-08 | 0.26 | 0.6031 | 0.3217 |
| rs7563777 | 2 | 137512418 | C | T | 0.515968 | -0.0134158 | 0.00206241 | 7.80E-11 | 0.0109999 | 0.1442 | 0.0109701 |
| rs7652234 | 3 | 24190459 | G | C | 0.041119 | 0.0375649 | 0.00526303 | 9.50E-13 | 0.84 | 0.964 | 0.2047 |
| rs7727787 | 5 | 180653039 | A | G | 0.134618 | -0.0191551 | 0.00302872 | 2.50E-10 | 0.12 | 0.2305 | 0.732 |
| rs7742131 | 6 | 100723855 | G | T | 0.290245 | -0.0124536 | 0.00227045 | 4.10E-08 | 0.59 | 0.0902693 | 0.8338 |
| rs78352137 | 14 | 100920416 | C | T | 0.111605 | -0.0316275 | 0.00328354 | 5.90E-22 | 0.28 | 0.2711 | 0.0630507 |
| rs7852169 | 9 | 114318394 | G | C | 0.08261 | 0.0455731 | 0.00375283 | 6.20E-34 | 0.0619998 | 0.0154301 | 0.5196 |
| rs7853970 | 9 | 86715566 | C | T | 0.536328 | -0.019012 | 0.00209114 | 9.80E-20 | 0.14 | 0.1127 | 0.1426 |
| rs7854743 | 9 | 75943800 | T | A | 0.063947 | 0.026527 | 0.00421738 | 3.20E-10 | 0.0580003 | 0.3983 | 0.9553 |
| rs7915178 | 10 | 1734473 | C | T | 0.548323 | 0.0184815 | 0.00210951 | 1.90E-18 | 0.74 | 0.3063 | 0.0121899 |
| rs80170948 | 5 | 64020316 | G | T | 0.042523 | 0.0294043 | 0.00535288 | 3.90E-08 | 0.26 | 0.8551 | 0.0678203 |
| rs8112411 | 19 | 9991735 | A | G | 0.625022 | 0.0235928 | 0.00213323 | 2.00E-28 | 0.016 | 0.0448497 | 0.02495 |
| rs815715 | 3 | 61264084 | G | C | 0.425741 | 0.0116583 | 0.0020966 | 2.70E-08 | * | * | * |
| rs842548 | 2 | 184262798 | C | A | 0.799987 | -0.0145504 | 0.00258552 | 1.80E-08 | 0.0269998 | 0.1599 | 0.4877 |
| rs852033 | 20 | 17085493 | C | A | 0.752402 | 0.0163911 | 0.00239562 | 7.80E-12 | 0.22 | 0.03476 | 0.705601 |
| rs913588 | 9 | 7174673 | A | G | 0.51535 | -0.0186095 | 0.0020634 | 1.90E-19 | 0.98 | 0.7538 | 0.0780405 |
| rs9322076 | 6 | 100952020 | T | C | 0.551428 | -0.0186098 | 0.00208389 | 4.20E-19 | 0.0290001 | 0.6167 | 0.9425 |
| rs9341625 | 6 | 77709617 | T | C | 0.20543 | -0.0233803 | 0.00255099 | 4.90E-20 | 0.98 | 0.0415997 | 0.128 |
| rs9397414 | 6 | 151806283 | G | A | 0.316362 | -0.0136894 | 0.0022207 | 7.10E-10 | 0.95 | 0.3567 | 0.4968 |
| rs9542595 | 13 | 71722247 | A | G | 0.308715 | 0.013156 | 0.0022491 | 4.90E-09 | 0.11 | 0.0809096 | 0.1636 |
| rs9568117 | 13 | 49452803 | C | A | 0.159945 | 0.0188351 | 0.0028238 | 2.60E-11 | 0.31 | 0.2738 | 0.8044 |
| rs9592952 | 13 | 74525794 | C | A | 0.41909 | 0.0132879 | 0.00209635 | 2.30E-10 | 0.0280001 | 0.0446098 | 0.9106 |
| rs9594366 | 13 | 40368601 | T | C | 0.350836 | 0.0148672 | 0.00217086 | 7.50E-12 | 1 | 0.2826 | 0.669501 |
| rs9616551 | 22 | 49680429 | G | A | 0.802113 | -0.0158305 | 0.00260853 | 1.30E-09 | 0.85 | 0.372 | 0.753401 |
| rs9635759 | 17 | 49613785 | A | G | 0.303865 | 0.0273668 | 0.00229454 | 8.60E-33 | 0.35 | 0.1862 | 0.9111 |
| rs9696767 | 9 | 92515702 | G | A | 0.406705 | -0.0122001 | 0.00212144 | 8.90E-09 | 0.66 | 0.7633 | 0.2803 |
| rs9757252 | 3 | 86894478 | C | T | 0.617131 | 0.0208834 | 0.00212675 | 9.30E-23 | 0.67 | 0.885 | 0.1973 |
| rs9790189 | 3 | 117583647 | A | T | 0.498034 | 0.0229027 | 0.00206782 | 1.60E-28 | * | # | * |
| rs9816563 | 3 | 24702344 | C | T | 0.501639 | 0.0127161 | 0.0020645 | 7.30E-10 | 0.27 | 0.704601 | 0.4947 |
| rs9849338 | 3 | 172126476 | A | C | 0.35963 | -0.0146123 | 0.00216509 | 1.50E-11 | 0.0299999 | 0.0386598 | # |
| rs9851777 | 3 | 88267467 | C | T | 0.884106 | -0.0236166 | 0.00321381 | 2.00E-13 | 0.450001 | 0.3066 | 0.1772 |
| Number of SNPs | | | | | | | | | 194 | 194 | 193 |
| F-statistics | | | | | | | | | 67.80 | 67.84 | 68.09 |
| SNP | Chr | Position | Effect allele | Other allele | EAF | Exposure (AMP) | | | Outcome (P value) | | |
|  |  |  |  |  |  | Beta | SE | P value | Overall OA | Knee OA | Hip OA |
| rs1020622 | 11 | 9469792 | G | C | 0.425046 | 0.0218648 | 0.00373281 | 4.70E-09 | * | * | * |
| rs10476835 | 5 | 141675985 | A | G | 0.48716 | 0.0211285 | 0.00369077 | 1.00E-08 | 0.0860003 | 0.0361202 | 0.503499 |
| rs10521305 | 16 | 53908484 | C | T | 0.059401 | 0.0543913 | 0.00778033 | 2.70E-12 | 0.37 | 0.0773695 | 0.3987 |
| rs10813912 | 9 | 32975210 | G | A | 0.623209 | 0.0248784 | 0.00380188 | 6.00E-11 | 0.6 | 0.2961 | 0.664 |
| rs10854167 | 20 | 61533039 | C | G | 0.21285 | -0.0394814 | 0.0045064 | 1.90E-18 | 0.74 | 0.2525 | 0.499401 |
| rs10937153 | 3 | 183562925 | A | G | 0.240679 | 0.0360705 | 0.00432513 | 7.40E-17 | 0.31 | 0.6336 | 0.6691 |
| rs10998203 | 10 | 70224532 | G | C | 0.203589 | -0.0360969 | 0.00458046 | 3.30E-15 | 0.49 | 0.396 | 0.1026 |
| rs11031005 | 11 | 30226356 | C | T | 0.143527 | 0.0545996 | 0.00527248 | 3.90E-25 | 0.44 | 0.542 | 0.3197 |
| rs112190116 | 7 | 144096158 | T | C | 0.011146 | 0.142691 | 0.0175968 | 5.10E-16 | 0.69 | 0.1765 | 0.1584 |
| rs112217463 | 15 | 41438563 | A | G | 0.373637 | -0.0373438 | 0.00382293 | 1.50E-22 | 0.67 | 0.9717 | 0.6324 |
| rs11571818 | 13 | 32968810 | C | T | 0.009415 | -0.112885 | 0.0190859 | 3.30E-09 | # | 0.8283 | 0.1419 |
| rs11650324 | 17 | 5330186 | G | A | 0.223193 | 0.0390919 | 0.00443178 | 1.10E-18 | 0.34 | 0.6369 | 0.6086 |
| rs12046563 | 1 | 43137280 | G | A | 0.239078 | -0.0251841 | 0.0043189 | 5.50E-09 | 0.81 | 0.3936 | 0.4617 |
| rs12132692 | 1 | 169732743 | T | C | 0.095686 | 0.0402635 | 0.00626821 | 1.30E-10 | 0.55 | 0.5006 | 0.745501 |
| rs12444283 | 16 | 33892028 | G | C | 0.495379 | -0.0289439 | 0.00396012 | 2.70E-13 | * | * | * |
| rs12503643 | 4 | 185746088 | T | G | 0.397102 | 0.0467221 | 0.00376808 | 2.60E-35 | 0.0259998 | 0.1009 | 0.8104 |
| rs12609254 | 19 | 46865945 | T | C | 0.10754 | -0.0338934 | 0.00594874 | 1.20E-08 | 0.9 | 0.774801 | 0.2274 |
| rs156520 | 4 | 155690081 | A | C | 0.285356 | 0.0224571 | 0.0041173 | 4.90E-08 | 0.450001 | 0.6857 | 0.6699 |
| rs1565909 | 4 | 84400330 | T | C | 0.4874 | 0.0623334 | 0.00368522 | 3.50E-64 | 0.22 | 0.5751 | 0.6078 |
| rs1655907 | 6 | 29918587 | C | T | 0.844827 | -0.0290465 | 0.00508668 | 1.10E-08 | 0.780001 | 0.4353 | 0.0204301 |
| rs16991615 | 20 | 5948227 | A | G | 0.061435 | 0.243363 | 0.00766417 | 1.00E-200 | 0.69 | 0.1332 | 0.687 |
| rs1760940 | 14 | 20938251 | C | A | 0.249675 | -0.0380724 | 0.00426482 | 4.40E-19 | 0.760001 | 0.8933 | 0.0612802 |
| rs17646517 | 3 | 44286015 | G | C | 0.023116 | -0.0715967 | 0.0122548 | 5.10E-09 | 0.0710003 | 0.583201 | 0.4122 |
| rs17650301 | 17 | 62479273 | C | A | 0.295896 | -0.0401265 | 0.00403659 | 2.80E-23 | 0.064 | 0.6726 | 0.4514 |
| rs17680522 | 16 | 9069331 | G | A | 0.292256 | 0.0231515 | 0.00406147 | 1.20E-08 | 0.94 | 0.0417196 | 0.4326 |
| rs17820747 | 2 | 32701687 | C | A | 0.237559 | 0.0259694 | 0.00433713 | 2.10E-09 | 0.77 | 0.714399 | 0.0400701 |
| rs17856037 | 14 | 34985658 | T | C | 0.025275 | -0.0739792 | 0.0117329 | 2.90E-10 | 0.75 | 0.52 | 0.5457 |
| rs1790123 | 12 | 123659542 | T | C | 0.80041 | 0.0346952 | 0.00460161 | 4.70E-14 | 2.00E-04 | 3.70E-09 | 0.4298 |
| rs184540366 | 12 | 66649478 | T | G | 0.022361 | -0.0848094 | 0.012884 | 4.60E-11 | 0.22 | 0.7121 | 0.673701 |
| rs200448 | 1 | 6701978 | C | T | 0.568616 | -0.0214999 | 0.00372895 | 8.10E-09 | 0.11 | 0.6821 | 0.0139499 |
| rs2241522 | 15 | 42127734 | G | A | 0.338832 | -0.0216094 | 0.0038967 | 2.90E-08 | 0.9 | 0.4507 | 0.4496 |
| rs2277339 | 12 | 57146069 | G | T | 0.106126 | -0.0780582 | 0.00599615 | 9.70E-39 | 0.56 | 0.0193001 | 0.2641 |
| rs2304192 | 19 | 3933314 | G | A | 0.674873 | -0.0281824 | 0.00404022 | 3.00E-12 | 0.1 | 0.5662 | 0.503499 |
| rs251723 | 16 | 11953080 | C | G | 0.343901 | 0.0517082 | 0.00388425 | 2.00E-40 | 0.17 | 0.8717 | 0.3699 |
| rs2519673 | 7 | 105994726 | A | G | 0.631019 | -0.0267774 | 0.00381997 | 2.40E-12 | 0.67 | 0.1 | 0.8808 |
| rs2524119 | 6 | 31229404 | C | T | 0.548225 | 0.0237527 | 0.00368586 | 1.20E-10 | 0.46 | 0.0572598 | 0.249 |
| rs2624847 | 3 | 50174197 | T | G | 0.743348 | 0.0268408 | 0.00422243 | 2.10E-10 | 0.29 | 0.1331 | 0.4885 |
| rs2688194 | 5 | 154257868 | C | T | 0.083337 | 0.0440712 | 0.00665805 | 3.60E-11 | 0.92 | 0.5836 | 0.672901 |
| rs28416520 | 12 | 130823657 | A | G | 0.455502 | -0.0356415 | 0.00372893 | 1.20E-21 | 0.57 | 0.2616 | 0.3947 |
| rs2844466 | 6 | 31601012 | C | T | 0.358718 | -0.0472066 | 0.00383583 | 8.30E-35 | 0.58 | 0.2729 | 0.1386 |
| rs28797500 | 8 | 37884310 | C | T | 0.777853 | -0.0846069 | 0.00443452 | 3.80E-81 | 0.25 | 0.4633 | 0.5218 |
| rs2941506 | 17 | 37833035 | G | A | 0.684301 | 0.0373779 | 0.00397463 | 5.20E-21 | 0.450001 | 0.9717 | 0.457 |
| rs299168 | 19 | 56317058 | A | G | 0.097587 | 0.0506456 | 0.00628843 | 8.00E-16 | 0.12 | 0.0893408 | 0.8 |
| rs345985 | 3 | 156397174 | T | C | 0.41955 | -0.0238739 | 0.00374945 | 1.90E-10 | 0.00460002 | 0.00993002 | 0.2273 |
| rs34811474 | 4 | 25408838 | A | G | 0.231433 | -0.0238895 | 0.0043662 | 4.50E-08 | 0.00109999 | 2.92E-05 | 0.1739 |
| rs349306 | 19 | 950694 | A | G | 0.871745 | 0.0525066 | 0.00561478 | 8.60E-21 | 0.92 | 0.2279 | 0.8316 |
| rs34962991 | 19 | 55827175 | A | G | 0.363977 | -0.0983543 | 0.00383238 | 2.90E-145 | 0.12 | 0.00368502 | 0.584 |
| rs3803756 | 17 | 55363674 | T | A | 0.640107 | -0.0247507 | 0.00384048 | 1.20E-10 | 0.15 | 0.253 | 0.9564 |
| rs394448 | 19 | 56452144 | C | G | 0.515449 | -0.020456 | 0.00370034 | 3.20E-08 | * | * | * |
| rs4049337 | 7 | 56162172 | C | G | 0.698593 | -0.0257386 | 0.00401737 | 1.50E-10 | 0.79 | 0.0348297 | 0.8224 |
| rs419128 | 5 | 6739791 | A | G | 0.346747 | -0.0347717 | 0.00386202 | 2.20E-19 | 0.24 | 0.0870202 | 0.696599 |
| rs4408133 | 1 | 242049649 | C | G | 0.334777 | 0.0316029 | 0.00391465 | 6.90E-16 | 0.52 | 0.8279 | 0.7807 |
| rs4491723 | 2 | 152280246 | G | A | 0.26152 | 0.0310704 | 0.00418072 | 1.10E-13 | 0.82 | 0.0350002 | 0.5943 |
| rs4495657 | 1 | 180949131 | C | T | 0.59515 | 0.0328462 | 0.00376043 | 2.40E-18 | 0.0021 | 0.132 | 0.3783 |
| rs4668354 | 2 | 171814750 | G | C | 0.624972 | 0.0347835 | 0.00380081 | 5.60E-20 | 0.21 | 0.1237 | 0.9438 |
| rs4679121 | 3 | 126160601 | T | C | 0.104812 | -0.038058 | 0.00604834 | 3.10E-10 | 0.15 | 0.3373 | 0.2104 |
| rs4716056 | 6 | 16278390 | G | A | 0.373622 | 0.0214312 | 0.00379142 | 1.60E-08 | 0.46 | 0.6709 | 0.9131 |
| rs4782369 | 16 | 88531861 | C | G | 0.325618 | 0.0300142 | 0.00392749 | 2.10E-14 | 0.77 | 0.8467 | 0.0814592 |
| rs4821798 | 22 | 39021165 | C | T | 0.307942 | -0.0434503 | 0.00398813 | 1.20E-27 | 0.55 | 0.779399 | 0.000675896 |
| rs4886238 | 13 | 61113739 | A | G | 0.327255 | 0.0379851 | 0.0039464 | 6.30E-22 | 0.96 | 0.9396 | 0.8892 |
| rs507926 | 3 | 9008174 | C | T | 0.833471 | 0.0298505 | 0.00494009 | 1.50E-09 | 0.58 | 0.7558 | 0.1389 |
| rs536092 | 20 | 48501351 | T | C | 0.382668 | 0.0238492 | 0.00381037 | 3.90E-10 | 0.21 | 0.2937 | 0.0340298 |
| rs55707872 | 1 | 46680167 | C | A | 0.314362 | 0.0353746 | 0.00397578 | 5.70E-19 | 0.44 | 0.1113 | 0.2491 |
| rs55848327 | 15 | 63819427 | A | G | 0.768098 | 0.0251522 | 0.00437233 | 8.80E-09 | 0.86 | 0.580399 | 0.7611 |
| rs58279426 | 5 | 176448139 | C | T | 0.478628 | 0.0675842 | 0.00369987 | 1.50E-74 | 0.26 | 0.0610506 | 0.7326 |
| rs6011452 | 20 | 61289445 | A | C | 0.221177 | -0.0490561 | 0.00444771 | 2.80E-28 | 0.41 | 0.715801 | 0.7278 |
| rs60907808 | 19 | 23104068 | G | A | 0.143874 | -0.0443796 | 0.00525887 | 3.20E-17 | 0.31 | 0.4014 | 0.3401 |
| rs6139074 | 20 | 63244 | C | A | 0.206738 | -0.0306464 | 0.00456118 | 1.80E-11 | 0.67 | 0.8577 | 0.6915 |
| rs61870304 | 10 | 126680627 | G | A | 0.114382 | -0.0368462 | 0.00611348 | 1.70E-09 | 0.5 | 0.6354 | 0.2999 |
| rs62156695 | 2 | 67649768 | G | A | 0.103904 | -0.0640509 | 0.00603671 | 2.70E-26 | 0.709999 | 0.5675 | 0.0629898 |
| rs62445870 | 7 | 50514904 | T | C | 0.022584 | 0.0745712 | 0.012408 | 1.90E-09 | 0.93 | 0.5292 | 0.8618 |
| rs6435156 | 2 | 203425475 | T | C | 0.255236 | -0.0263107 | 0.0042185 | 4.50E-10 | 3.60E-05 | 0.0136201 | 0.781399 |
| rs6470643 | 8 | 129621777 | C | A | 0.217487 | -0.0282893 | 0.0044663 | 2.40E-10 | 0.16 | 0.0672001 | 0.0252 |
| rs6500437 | 16 | 89789898 | C | T | 0.315452 | -0.0354601 | 0.00397468 | 4.60E-19 | 0.58 | 0.03211 | 0.7264 |
| rs6584351 | 10 | 101970997 | G | A | 0.511463 | 0.0204696 | 0.00367696 | 2.60E-08 | 0.0790005 | 0.8238 | 0.344 |
| rs6667957 | 1 | 244613791 | C | T | 0.43005 | -0.0259762 | 0.00374996 | 4.30E-12 | 0.98 | 0.9155 | 0.0673194 |
| rs6736096 | 2 | 216958369 | C | T | 0.51589 | 0.0201113 | 0.00368295 | 4.70E-08 | 0.780001 | 0.790801 | 0.8208 |
| rs6830848 | 4 | 48690408 | T | G | 0.469038 | -0.0347717 | 0.00368223 | 3.60E-21 | 0.709999 | 0.4127 | 0.4533 |
| rs6912979 | 6 | 160120799 | C | T | 0.28336 | -0.0255493 | 0.00409197 | 4.30E-10 | 0.97 | 0.4552 | 0.5538 |
| rs6961014 | 7 | 128692710 | G | C | 0.787705 | -0.029044 | 0.00451308 | 1.20E-10 | 0.56 | 0.6534 | 0.3408 |
| rs7087644 | 10 | 97826334 | G | A | 0.040862 | -0.0839477 | 0.00931223 | 2.00E-19 | 0.6 | 0.701501 | 0.8166 |
| rs7125555 | 11 | 32549463 | T | C | 0.484015 | -0.0276469 | 0.00368739 | 6.50E-14 | 0.52 | 0.272 | 0.3005 |
| rs72708144 | 1 | 149815740 | C | T | 0.041604 | 0.0587965 | 0.00921141 | 1.70E-10 | 0.33 | 0.1288 | 0.3115 |
| rs72814771 | 5 | 173471004 | G | T | 0.09862 | 0.0493593 | 0.0062528 | 2.90E-15 | 0.51 | 0.5926 | 0.7743 |
| rs72827480 | 2 | 121146501 | C | T | 0.402064 | 0.0270835 | 0.00376142 | 6.00E-13 | 0.93 | 0.339 | 0.6019 |
| rs728900 | 10 | 131590300 | A | T | 0.422089 | -0.0317053 | 0.00375738 | 3.20E-17 | * | * | * |
| rs73037453 | 19 | 33464225 | T | C | 0.190997 | -0.0264143 | 0.00468736 | 1.70E-08 | 0.0629999 | 0.3413 | 0.3187 |
| rs746748 | 20 | 25282967 | T | C | 0.068678 | 0.0474397 | 0.00728581 | 7.50E-11 | 0.00589997 | 0.8763 | 0.6807 |
| rs74701710 | 10 | 13208912 | A | G | 0.056067 | -0.0483435 | 0.00803605 | 1.80E-09 | 0.41 | 0.7097 | 0.5244 |
| rs74742883 | 7 | 99785765 | G | T | 0.218286 | -0.0310808 | 0.00445473 | 3.00E-12 | 0.26 | 0.8166 | 0.6147 |
| rs75770066 | 12 | 66704225 | G | A | 0.030813 | 0.231048 | 0.0106265 | 8.09E-105 | 0.36 | 0.5507 | 0.771999 |
| rs7589040 | 2 | 135682208 | T | C | 0.209818 | -0.0315167 | 0.00449719 | 2.40E-12 | 0.8 | 0.3402 | 0.0804192 |
| rs7661090 | 4 | 13571901 | T | C | 0.106995 | -0.0352894 | 0.0059614 | 3.20E-09 | 0.0239999 | 0.2331 | 0.785301 |
| rs76928871 | 2 | 48005821 | G | A | 0.192314 | 0.043013 | 0.00468506 | 4.30E-20 | 0.47 | 0.7117 | 0.00388902 |
| rs77100210 | 12 | 10875928 | C | A | 0.051115 | 0.113128 | 0.008439 | 5.60E-41 | 0.83 | 0.4721 | 0.6003 |
| rs7778113 | 7 | 5448087 | T | G | 0.345864 | 0.0279687 | 0.0039179 | 9.40E-13 | 0.15 | 0.539299 | 0.9591 |
| rs7779 | 1 | 246930564 | C | G | 0.072772 | 0.0398625 | 0.00707203 | 1.70E-08 | 0.08 | 0.8268 | 0.00326498 |
| rs77952879 | 8 | 103636045 | C | G | 0.078277 | -0.0421247 | 0.00694851 | 1.30E-09 | 0.760001 | 0.6785 | 0.3237 |
| rs78080415 | 6 | 111537016 | C | T | 0.144619 | 0.0298435 | 0.00524617 | 1.30E-08 | 0.79 | 0.00389798 | 0.0237799 |
| rs7827991 | 8 | 81754701 | A | C | 0.078386 | 0.041731 | 0.00688397 | 1.30E-09 | 0.12 | 0.9923 | 0.01649 |
| rs78385274 | 2 | 67531571 | C | G | 0.105585 | -0.0593868 | 0.00600442 | 4.60E-23 | * | 0.5058 | 0.1232 |
| rs78385274 | 2 | 67531571 | C | G | 0.105585 | -0.0593868 | 0.00600442 | 4.60E-23 | 0.11 | * | * |
| rs7845046 | 8 | 48926264 | A | T | 0.948961 | -0.0492105 | 0.0083836 | 4.40E-09 | 0.79 | 0.3341 | 0.0345096 |
| rs8045589 | 16 | 9210373 | T | A | 0.486707 | -0.0225953 | 0.0036951 | 9.70E-10 | * | * | * |
| rs809673 | 2 | 27656036 | G | A | 0.397602 | -0.0417213 | 0.00376875 | 1.70E-28 | 0.79 | 0.0795903 | 0.2078 |
| rs888368 | 15 | 86302283 | G | A | 0.505234 | 0.0221884 | 0.00368454 | 1.70E-09 | 0.92 | 0.4278 | 0.6857 |
| rs9307242 | 4 | 100934530 | C | T | 0.354717 | -0.0271568 | 0.00382072 | 1.20E-12 | 0.14 | 0.4425 | 0.5695 |
| rs9313736 | 5 | 175948316 | A | G | 0.385808 | -0.0314315 | 0.00377284 | 8.00E-17 | 0.38 | 0.8249 | 0.7061 |
| rs9358956 | 6 | 10887253 | C | G | 0.828667 | -0.0658709 | 0.00494254 | 1.60E-40 | 0.9 | 0.9264 | 0.8074 |
| rs9438982 | 1 | 39358143 | A | C | 0.681683 | -0.0471437 | 0.00394883 | 7.40E-33 | 0.68 | 0.7891 | 0.1001 |
| rs9607474 | 22 | 22336708 | T | C | 0.117784 | -0.032426 | 0.00573242 | 1.50E-08 | 0.43 | 0.0973509 | 0.8575 |
| rs9613667 | 22 | 29127412 | C | A | 0.348026 | -0.0232454 | 0.00387681 | 2.00E-09 | 0.33 | 0.1349 | 0.0002016 |
| rs9788714 | 15 | 89781855 | A | G | 0.381174 | -0.0429278 | 0.00380771 | 1.80E-29 | 0.29 | 0.9227 | 0.2888 |
| rs9818740 | 3 | 135939586 | A | G | 0.274865 | -0.0278441 | 0.00413884 | 1.70E-11 | 0.64 | 0.00826 | 0.6871 |
| rs9915489 | 17 | 41173226 | T | A | 0.3405 | 0.043559 | 0.00390689 | 7.20E-29 | 0.9 | 0.0196698 | 0.7149 |
| Number of SNPs | | | | | | | | | 108 | 109 | 109 |
| F-statistics | | | | | | | | | 95.39 | 94.88 | 94.88 |
| SNP | Chr | Position | Effect allele | Other allele | EAF | Exposure (AFB) | | | Outcome (P value) | | |
|  |  |  |  |  |  | Beta | SE | P value | Overall OA | Knee OA | Hip OA |
| rs10922907 | 1 | 91193049 | T | A | 0.549966 | 0.0229573 | 0.00396508 | 7.06E-09 | * | * | * |
| rs11669516 | 19 | 19532682 | A | G | 0.186338 | -0.0286249 | 0.00503958 | 1.35E-08 | 0.01 | 0.641899 | 0.2248 |
| rs11743711 | 5 | 60735530 | C | T | 0.391892 | -0.0221391 | 0.00403687 | 4.16E-08 | 0.77 | 0.1595 | 0.0773393 |
| rs12435203 | 14 | 103225591 | A | G | 0.296608 | -0.023764 | 0.00432802 | 4.01E-08 | 0.87 | 0.0201799 | 0.769501 |
| rs13437378 | 6 | 14507185 | C | T | 0.142434 | -0.0315484 | 0.00577861 | 4.78E-08 | 0.15 | 0.4974 | 0.8379 |
| rs1449475 | 2 | 142821525 | A | T | 0.54139 | 0.0222356 | 0.00395464 | 1.88E-08 | * | * | * |
| rs2230590 | 3 | 49936102 | C | T | 0.517564 | -0.0331541 | 0.00394007 | 3.98E-17 | 0.0189998 | 6.23E-05 | 0.0259597 |
| rs28409139 | 2 | 60514540 | C | T | 0.591308 | -0.0264983 | 0.00401724 | 4.24E-11 | 0.36 | 0.680899 | 0.2153 |
| rs34310332 | 2 | 104225533 | T | A | 0.0926856 | 0.0408768 | 0.00710887 | 8.94E-09 | 0.3 | 0.6748 | 0.6011 |
| rs362307 | 4 | 3241845 | T | C | 0.0760278 | -0.0432296 | 0.00749946 | 8.22E-09 | 0.17 | 0.1142 | 0.2942 |
| rs55773130 | 7 | 2070554 | C | T | 0.179938 | 0.0295907 | 0.00512769 | 7.91E-09 | 0.12 | 0.00857808 | 0.2042 |
| rs6722254 | 2 | 174032551 | G | A | 0.625939 | 0.0229281 | 0.00405943 | 1.63E-08 | 0.719999 | 0.1014 | 0.5061 |
| rs7046483 | 9 | 14777395 | A | G | 0.609561 | 0.0224356 | 0.00404948 | 3.02E-08 | 0.95 | 0.5583 | 0.5641 |
| rs7321696 | 13 | 67037493 | T | C | 0.139833 | -0.0317974 | 0.00575181 | 3.24E-08 | 0.0379997 | 0.0544102 | 0.0307001 |
| rs7815125 | 8 | 87680112 | A | T | 0.824847 | 0.0293895 | 0.00517501 | 1.36E-08 | 0.68 | 0.7822 | 0.9056 |
| rs7830431 | 8 | 10700317 | G | A | 0.59843 | 0.0222622 | 0.00402698 | 3.24E-08 | 0.32 | 0.00631001 | 0.00301002 |
| rs8059365 | 16 | 76252388 | C | A | 0.441291 | -0.0221266 | 0.00396233 | 2.35E-08 | 0.2 | 0.2306 | 0.9037 |
| rs896686 | 18 | 53123031 | G | T | 0.173028 | 0.0294932 | 0.00526268 | 2.10E-08 | 0.0560003 | 0.2714 | 0.0527801 |
| rs9372625 | 6 | 98344031 | A | G | 0.381688 | 0.0230014 | 0.00406662 | 1.55E-08 | 0.94 | 0.0754606 | 0.8276 |
| Number of SNPs | | | | | | | | | 17 | 17 | 17 |
| F-statistics | | | | | | | | | 32.34 | 32.34 | 32.34 |
| SNP | Chr | Position | Effect allele | Other allele | EAF | Exposure (ALB) | | | Outcome (P value) | | |
|  |  |  |  |  |  | Beta | SE | P value | Overall OA | Knee OA | Hip OA |
| rs10964737 | 9 | 2093264 | C | T | 0.01222 | 0.0863379 | 0.0155676 | 2.90E-08 | 0.54 | 0.3803 | 0.5887 |
| rs12250380 | 10 | 106587950 | G | A | 0.5825 | 0.0201909 | 0.00343666 | 4.20E-09 | 0.0920005 | 0.549301 | 0.5359 |
| rs1267490 | 6 | 14717463 | A | G | 0.814111 | -0.024378 | 0.00435317 | 2.10E-08 | 0.98 | 0.0097499 | 0.9282 |
| rs2208082 | 20 | 14761085 | A | G | 0.568997 | 0.0190597 | 0.0034371 | 2.90E-08 | 0.37 | 0.1221 | 0.2533 |
| rs359253 | 2 | 60481956 | A | G | 0.639341 | -0.0222912 | 0.00352058 | 2.40E-10 | 0.55 | 0.6623 | 0.2248 |
| rs6446187 | 3 | 49907111 | A | C | 0.512378 | -0.0291866 | 0.0033823 | 6.20E-18 | 0.0350002 | 0.000127201 | 0.0259502 |
| Number of SNPs | | | | | | | | | 6 | 6 | 6 |
| F-statistics | | | | | | | | | 40.37 | 40.37 | 40.37 |
| SNP | Chr | Position | Effect allele | Other allele | EAF | Exposure (NLB) | | | Outcome (P value) | | |
|  |  |  |  |  |  | Beta | SE | P value | Overall OA | Knee OA | Hip OA |
| rs10749233 | 10 | 118777998 | C | G | 0.75947 | -0.0253112 | 0.00454895 | 2.64E-08 | 0.95 | 0.2164 | 0.6406 |
| rs4869737 | 6 | 151892135 | T | C | 0.701393 | -0.0294352 | 0.00419316 | 2.23E-12 | 0.064 | 0.8624 | 0.6126 |
| rs7041058 | 9 | 3022270 | G | T | 0.521624 | 0.0212204 | 0.00386054 | 3.87E-08 | 0.0490004 | 0.4174 | 0.0689795 |
| Number of SNPs | | | | | | | | | 3 | 3 | 3 |
| F-statistics | | | | | | | | | 36.83 | 36.83 | 36.83 |
| SNP | Chr | Position | Effect allele | Other allele | EAF | Exposure (AFSI) | | | Outcome (P value) | | |
|  |  |  |  |  |  | Beta | SE | P value | Overall OA | Knee OA | Hip OA |
| rs1000586 | 5 | 76938952 | C | T | 0.356215 | -0.0129893 | 0.00227885 | 1.20E-08 | 0.64 | 0.487401 | 0.512 |
| rs10085776 | 7 | 104441909 | C | T | 0.565458 | -0.013742 | 0.00218111 | 3.00E-10 | 0.709999 | 0.768599 | 0.8322 |
| rs10104523 | 8 | 73889570 | C | T | 0.522362 | -0.0125544 | 0.00216093 | 6.30E-09 | 0.85 | 0.1206 | 0.609101 |
| rs10137475 | 14 | 58797953 | A | G | 0.577345 | 0.0132976 | 0.00218374 | 1.10E-09 | 0.0619998 | 0.3634 | 0.8838 |
| rs10144067 | 14 | 93885198 | T | C | 0.591023 | -0.0141806 | 0.00221301 | 1.50E-10 | 0.61 | 0.0239299 | 0.5675 |
| rs10204104 | 2 | 174209822 | A | C | 0.831892 | -0.0163744 | 0.00289388 | 1.50E-08 | 0.14 | 0.605399 | 0.9006 |
| rs10233473 | 7 | 32265007 | T | C | 0.252363 | -0.0144439 | 0.00249914 | 7.50E-09 | 0.57 | 0.0190801 | 0.3593 |
| rs10492794 | 16 | 12639850 | T | C | 0.178188 | 0.0161576 | 0.00283198 | 1.20E-08 | 0.41 | 0.2876 | 0.00472498 |
| rs10496949 | 2 | 144153344 | C | G | 0.578036 | 0.0149311 | 0.00218449 | 8.20E-12 | * | * | * |
| rs10750367 | 11 | 127449675 | G | A | 0.554253 | -0.0152168 | 0.00218878 | 3.60E-12 | 0.33 | 0.4917 | 0.4616 |
| rs10880088 | 12 | 41905897 | T | C | 0.216086 | 0.0161646 | 0.00263079 | 8.00E-10 | 0.34 | 0.1363 | 0.735401 |
| rs10886022 | 10 | 118778079 | C | A | 0.763037 | 0.0177217 | 0.00258759 | 7.50E-12 | 0.97 | 0.1626 | 0.5619 |
| rs10922907 | 1 | 91193049 | T | A | 0.549415 | 0.0224624 | 0.00217004 | 4.10E-25 | * | * | * |
| rs10994777 | 10 | 63233988 | A | G | 0.161939 | 0.0188266 | 0.0029331 | 1.40E-10 | 0.49 | 0.5869 | 0.9525 |
| rs11030102 | 11 | 27681596 | G | C | 0.261081 | -0.0141577 | 0.00245584 | 8.20E-09 | 0.81 | 0.0599198 | 0.3513 |
| rs11030402 | 11 | 28676170 | G | A | 0.368192 | 0.0131312 | 0.00223892 | 4.50E-09 | 0.0870001 | 0.00101801 | 0.1506 |
| rs11111153 | 12 | 102476087 | C | A | 0.245107 | -0.0139553 | 0.00250685 | 2.60E-08 | 0.46 | 0.555801 | 0.2089 |
| rs11123817 | 2 | 100835032 | T | C | 0.401206 | 0.0150766 | 0.00219576 | 6.60E-12 | 0.760001 | 0.0341099 | 0.4718 |
| rs11188601 | 10 | 97856899 | C | T | 0.364739 | -0.0172077 | 0.00224022 | 1.60E-14 | 0.38 | 0.7582 | 0.7428 |
| rs112633616 | 12 | 23238326 | C | A | 0.033261 | 0.0403127 | 0.00606962 | 3.10E-11 | 0.51 | 0.7514 | 0.2467 |
| rs112780312 | 1 | 153797015 | A | G | 0.275094 | -0.0165258 | 0.00244083 | 1.30E-11 | 0.649999 | 0.9707 | 0.1595 |
| rs113338260 | 5 | 46004640 | C | T | 0.215732 | -0.0156495 | 0.00262476 | 2.50E-09 | 0.17 | 0.3514 | 0.00652695 |
| rs113367286 | 7 | 140144414 | T | C | 0.278201 | 0.0198309 | 0.00241196 | 2.00E-16 | 0.53 | 0.2758 | 0.6598 |
| rs1139897 | 16 | 720986 | A | G | 0.223525 | 0.0175687 | 0.00259324 | 1.20E-11 | 0.015 | 0.0508803 | 0.685499 |
| rs1156981 | 1 | 88829969 | G | A | 0.901596 | -0.0227567 | 0.00361129 | 2.90E-10 | 0.62 | 0.4391 | 0.1956 |
| rs115882849 | 3 | 54156724 | A | G | 0.152996 | -0.0191877 | 0.00300418 | 1.70E-10 | 0.27 | 0.3823 | 0.668099 |
| rs11678980 | 2 | 162101261 | A | G | 0.461083 | -0.0123757 | 0.00223207 | 2.90E-08 | 0.649999 | 0.0539896 | 0.3686 |
| rs11729080 | 4 | 112503872 | A | G | 0.171149 | 0.0229745 | 0.00285872 | 9.20E-16 | 0.16 | 0.0352298 | 0.3035 |
| rs11770163 | 7 | 116417848 | C | G | 0.331573 | -0.0130247 | 0.0022936 | 1.40E-08 | 0.27 | 0.4921 | 0.8049 |
| rs12147463 | 14 | 41059928 | A | G | 0.193433 | -0.020875 | 0.00275919 | 3.90E-14 | 0.21 | 0.713001 | 0.4411 |
| rs12204714 | 6 | 152235339 | T | C | 0.632102 | 0.0292636 | 0.00223744 | 4.30E-39 | 0.17 | 0.4457 | 0.0321899 |
| rs1226414 | 2 | 157109930 | T | A | 0.505107 | 0.0155399 | 0.00215833 | 6.00E-13 | * | * | * |
| rs12448731 | 16 | 49622284 | T | C | 0.141723 | -0.0174598 | 0.00310836 | 1.90E-08 | 0.34 | 0.3784 | 0.3609 |
| rs12468863 | 2 | 26940294 | T | C | 0.517874 | 0.0135539 | 0.00216892 | 4.10E-10 | 0.37 | 0.00286702 | 0.0858895 |
| rs12505942 | 4 | 140906390 | C | T | 0.342778 | 0.0160611 | 0.00227551 | 1.70E-12 | 0.1 | 0.0708696 | 0.281 |
| rs12511982 | 4 | 60736871 | A | G | 0.550535 | 0.0126559 | 0.00217493 | 5.90E-09 | 0.16 | 0.188 | 0.7508 |
| rs12523097 | 5 | 167411400 | C | T | 0.297727 | -0.0160994 | 0.00237205 | 1.10E-11 | 0.24 | 0.7927 | 0.758799 |
| rs12523398 | 5 | 45119647 | A | T | 0.173891 | 0.0225484 | 0.00285708 | 3.00E-15 | 0.064 | 0.2278 | 0.5201 |
| rs12653396 | 5 | 87847273 | A | T | 0.565389 | -0.0219752 | 0.00219358 | 1.30E-23 | * | * | * |
| rs12671608 | 7 | 153524115 | C | T | 0.139057 | -0.017507 | 0.00313092 | 2.20E-08 | 0.760001 | 0.0242198 | 0.4134 |
| rs12701263 | 7 | 32962089 | T | C | 0.400041 | 0.0144102 | 0.00220279 | 6.10E-11 | 0.3 | 0.2988 | 0.8414 |
| rs12795483 | 11 | 79883503 | G | A | 0.518144 | 0.0129681 | 0.00216666 | 2.20E-09 | 0.98 | 0.2535 | 0.03252 |
| rs12826060 | 12 | 24195419 | G | T | 0.412502 | -0.0130686 | 0.00219153 | 2.50E-09 | 0.0790005 | 0.2852 | 0.0230001 |
| rs12907546 | 15 | 47684280 | A | G | 0.211749 | -0.0228102 | 0.00264994 | 7.40E-18 | 0.47 | 0.4793 | 0.935 |
| rs1295220 | 5 | 154890837 | T | C | 0.25348 | -0.0186416 | 0.00248775 | 6.70E-14 | 0.44 | 0.2072 | 0.6147 |
| rs12970816 | 18 | 50023859 | A | G | 0.395202 | -0.0152412 | 0.00221166 | 5.50E-12 | 0.709999 | 0.8258 | 0.3526 |
| rs13009008 | 2 | 174043233 | G | A | 0.672296 | 0.0182638 | 0.0022916 | 1.60E-15 | 0.57 | 0.0925209 | 0.7642 |
| rs13289229 | 9 | 86224419 | T | G | 0.178174 | 0.0178001 | 0.00285474 | 4.50E-10 | 0.74 | 0.0776605 | 0.881 |
| rs1368546 | 2 | 104057364 | C | T | 0.55706 | 0.015985 | 0.00216603 | 1.60E-13 | 0.64 | 0.3745 | 0.8074 |
| rs1391075 | 12 | 84052203 | C | A | 0.643432 | -0.0153773 | 0.00225175 | 8.50E-12 | 0.56 | 0.1856 | 0.8188 |
| rs1392816 | 1 | 66481188 | T | C | 0.387907 | 0.0175184 | 0.00222824 | 3.80E-15 | 0.91 | 0.09579 | 0.3223 |
| rs140098 | 22 | 30126644 | C | T | 0.547765 | -0.0123554 | 0.00218166 | 1.50E-08 | 0.0129999 | 0.001102 | 0.5312 |
| rs141547796 | 6 | 50615935 | A | G | 0.082193 | 0.0334614 | 0.00395855 | 2.80E-17 | 0.82 | 0.5316 | 0.8322 |
| rs1454687 | 3 | 94038085 | G | C | 0.514884 | 0.0119775 | 0.00215579 | 2.80E-08 | * | * | * |
| rs1547351 | 8 | 36842153 | A | T | 0.410447 | 0.0122226 | 0.00219383 | 2.50E-08 | 0.98 | 0.3146 | 0.704601 |
| rs1585634 | 8 | 54396376 | C | G | 0.802142 | -0.0155686 | 0.00272036 | 1.00E-08 | 0.74 | 0.0817994 | 0.00106699 |
| rs159428 | 20 | 31099311 | C | T | 0.526473 | -0.0133188 | 0.00216122 | 7.20E-10 | 0.57 | 0.9798 | 0.442 |
| rs16948048 | 17 | 47440466 | G | A | 0.36754 | -0.0166924 | 0.00223927 | 9.00E-14 | 0.49 | 0.2952 | 0.0773499 |
| rs17164088 | 4 | 2675893 | G | A | 0.422075 | -0.012374 | 0.0021824 | 1.40E-08 | 0.94 | 0.0228502 | 0.2583 |
| rs1812249 | 1 | 112284581 | A | G | 0.215227 | -0.0148834 | 0.00262062 | 1.40E-08 | 0.59 | 0.1431 | 0.5926 |
| rs182353 | 8 | 115396892 | T | C | 0.468089 | -0.013024 | 0.00216294 | 1.70E-09 | 0.11 | 0.9591 | 0.4849 |
| rs1891588 | 1 | 151597660 | C | G | 0.552814 | -0.0119638 | 0.00216512 | 3.30E-08 | * | * | * |
| rs1931263 | 1 | 96175101 | T | G | 0.4895 | -0.0121295 | 0.00215544 | 1.80E-08 | 0.77 | 0.2187 | 0.2734 |
| rs2025151 | 9 | 99161512 | G | C | 0.196133 | 0.0150046 | 0.00271665 | 3.30E-08 | 0.8 | 0.709999 | 0.5321 |
| rs206005 | 6 | 164427025 | T | A | 0.760199 | -0.0144473 | 0.00253165 | 1.20E-08 | 0.13 | 0.2223 | 0.2759 |
| rs2084572 | 3 | 17315758 | G | A | 0.450207 | 0.014725 | 0.00216537 | 1.00E-11 | 0.47 | 0.2897 | 0.0321004 |
| rs2093623 | 10 | 10922977 | A | G | 0.496285 | 0.0150008 | 0.00217668 | 5.50E-12 | 0.89 | 0.3068 | 0.3101 |
| rs2130893 | 10 | 134039132 | C | G | 0.285354 | 0.0149124 | 0.00246651 | 1.50E-09 | 0.43 | 0.7486 | 0.8834 |
| rs2174752 | 13 | 69332015 | T | G | 0.45124 | -0.0143345 | 0.00216954 | 3.90E-11 | 0.1 | 0.9773 | 0.295 |
| rs2176337 | 9 | 108959330 | T | A | 0.315293 | -0.017784 | 0.00232203 | 1.90E-14 | 0.46 | 0.8126 | 0.0241902 |
| rs2188151 | 3 | 50201924 | T | G | 0.424889 | -0.0222145 | 0.00218242 | 2.50E-24 | 0.0710003 | 8.50E-05 | 0.1693 |
| rs2274568 | 1 | 110612925 | A | G | 0.580677 | -0.0137862 | 0.00218862 | 3.00E-10 | 0.760001 | 5.75E-06 | 0.0706594 |
| rs2382440 | 9 | 14140575 | C | G | 0.155948 | -0.0173228 | 0.0029817 | 6.30E-09 | 0.23 | 0.579001 | 0.574 |
| rs239190 | 6 | 101129067 | T | C | 0.532174 | 0.0151426 | 0.00216441 | 2.60E-12 | 0.59 | 0.8955 | 0.5729 |
| rs2406374 | 5 | 106936435 | T | C | 0.314035 | 0.0147643 | 0.00231855 | 1.90E-10 | 0.9 | 0.2129 | 0.7319 |
| rs2535593 | 17 | 5621920 | C | T | 0.460207 | 0.0125197 | 0.00217263 | 8.30E-09 | 0.1 | 0.0283602 | 0.9606 |
| rs2553041 | 2 | 63238346 | C | G | 0.196229 | 0.0202801 | 0.00272704 | 1.00E-13 | 0.22 | 0.4145 | 0.913 |
| rs2612030 | 3 | 53773437 | C | T | 0.838125 | 0.0242699 | 0.00293365 | 1.30E-16 | 0.0269998 | 0.00211602 | 0.1796 |
| rs2613765 | 19 | 5066330 | A | G | 0.473384 | 0.01255 | 0.00216222 | 6.50E-09 | 0.0530005 | 0.9196 | 0.0178201 |
| rs2625223 | 1 | 204903874 | T | C | 0.48174 | 0.0124972 | 0.00218318 | 1.00E-08 | 0.004 | 0.1717 | 0.5416 |
| rs2744450 | 6 | 52951766 | G | A | 0.802128 | 0.017081 | 0.00270781 | 2.80E-10 | 0.709999 | 0.6742 | 0.2264 |
| rs293736 | 20 | 31925189 | C | A | 0.722176 | -0.0140549 | 0.00241272 | 5.70E-09 | 0.31 | 0.1687 | 0.9824 |
| rs2974311 | 8 | 42455166 | A | G | 0.497467 | 0.0138488 | 0.00215715 | 1.40E-10 | 0.48 | 0.0505801 | 0.6938 |
| rs298247 | 2 | 157233542 | C | G | 0.167472 | 0.016312 | 0.00288227 | 1.50E-08 | 0.0659994 | 0.4636 | 0.4921 |
| rs3007104 | 14 | 47367434 | A | G | 0.42401 | -0.0154973 | 0.00218798 | 1.40E-12 | 0.0290001 | 0.0681899 | 0.5528 |
| rs30266 | 5 | 103972357 | A | G | 0.328425 | -0.012649 | 0.00229698 | 3.70E-08 | 0.29 | 0.2841 | 0.6891 |
| rs341521 | 13 | 60399045 | A | G | 0.702818 | -0.0152941 | 0.00237256 | 1.10E-10 | 0.86 | 0.000167799 | 0.5774 |
| rs34517439 | 1 | 78450517 | A | C | 0.121862 | -0.0197888 | 0.00333274 | 2.90E-09 | 0.0719996 | 0.00128201 | 0.6955 |
| rs34606772 | 3 | 24908376 | T | C | 0.416265 | -0.0127837 | 0.00220229 | 6.40E-09 | 0.67 | 0.0259801 | 0.1275 |
| rs34811474 | 4 | 25408838 | A | G | 0.2308 | 0.015285 | 0.00255792 | 2.30E-09 | 0.00109999 | 2.92E-05 | 0.1739 |
| rs35128508 | 18 | 42722340 | A | G | 0.273405 | 0.0146939 | 0.00242383 | 1.30E-09 | 0.53 | 0.3363 | 0.6161 |
| rs35408390 | 2 | 86471782 | T | C | 0.44275 | -0.0121304 | 0.00216917 | 2.20E-08 | 0.28 | 0.0886707 | 0.9271 |
| rs35851551 | 7 | 31330785 | G | A | 0.101404 | -0.0264733 | 0.00360571 | 2.10E-13 | 0.0479999 | 0.7173 | 0.7193 |
| rs359239 | 2 | 60474600 | T | C | 0.573829 | -0.0189754 | 0.00219147 | 4.80E-18 | 1 | 0.3536 | 0.680201 |
| rs369230 | 16 | 89645437 | T | G | 0.692409 | -0.0184886 | 0.00235304 | 3.90E-15 | 0.16 | 0.1779 | 0.542099 |
| rs3739121 | 2 | 201171014 | C | G | 0.427116 | -0.0122648 | 0.00217575 | 1.70E-08 | * | * | * |
| rs3741499 | 12 | 56474379 | C | T | 0.649467 | -0.0161594 | 0.00226011 | 8.70E-13 | 0.66 | 0.00894396 | 0.683501 |
| rs3758790 | 11 | 105850090 | G | A | 0.359414 | 0.0153546 | 0.00225012 | 8.90E-12 | 0.85 | 0.4359 | 0.9211 |
| rs3789045 | 1 | 204586812 | T | C | 0.209024 | 0.0149923 | 0.00264802 | 1.50E-08 | 0.48 | 0.786799 | 0.8968 |
| rs3896224 | 10 | 106467853 | G | A | 0.414575 | 0.0213629 | 0.00220715 | 3.70E-22 | 0.15 | 0.4877 | 0.7134 |
| rs4075359 | 8 | 9487813 | C | T | 0.625175 | 0.0188718 | 0.0022331 | 2.90E-17 | 0.5 | 0.0233502 | 5.49E-06 |
| rs435538 | 5 | 24921398 | G | C | 0.230492 | -0.0178171 | 0.00255912 | 3.40E-12 | 0.0490004 | 0.7807 | 0.5919 |
| rs4443996 | 10 | 134191005 | C | A | 0.476487 | -0.0167341 | 0.002167 | 1.10E-14 | 0.68 | 0.0656493 | 0.5464 |
| rs4602427 | 3 | 117474457 | G | C | 0.803857 | -0.0162149 | 0.0027194 | 2.50E-09 | 0.0990011 | 0.8392 | 0.8131 |
| rs4702 | 15 | 91426560 | A | G | 0.556374 | 0.0167404 | 0.00217251 | 1.30E-14 | 0.47 | 0.1158 | 0.00941499 |
| rs4727799 | 7 | 114110568 | T | C | 0.647272 | -0.0208488 | 0.00225933 | 2.80E-20 | 0.00460002 | 0.0151401 | 0.5596 |
| rs4735438 | 8 | 97829917 | T | C | 0.587017 | -0.0144891 | 0.00219239 | 3.90E-11 | 0.75 | 0.4684 | 0.5868 |
| rs4755749 | 11 | 43865501 | G | A | 0.403067 | 0.0157811 | 0.00219972 | 7.30E-13 | 0.18 | 0.002142 | 0.6568 |
| rs4800204 | 18 | 22647270 | T | C | 0.570335 | -0.0127969 | 0.00218287 | 4.60E-09 | 0.760001 | 0.1995 | 0.05223 |
| rs4805761 | 19 | 32951800 | G | A | 0.843347 | 0.0165446 | 0.00297366 | 2.60E-08 | 0.08 | 0.2859 | 0.3199 |
| rs4873133 | 8 | 51127054 | T | C | 0.292431 | -0.0180907 | 0.00237345 | 2.50E-14 | 0.43 | 0.3193 | 0.604399 |
| rs4937872 | 11 | 112827715 | G | A | 0.407721 | -0.0160337 | 0.0022087 | 3.90E-13 | 0.26 | 0.6712 | 0.9446 |
| rs4952343 | 2 | 32858637 | G | A | 0.446361 | 0.0138227 | 0.00219423 | 3.00E-10 | 0.00329997 | 0.1549 | 0.1468 |
| rs4961705 | 9 | 16347927 | C | G | 0.348007 | 0.0134658 | 0.00230266 | 5.00E-09 | 0.0179999 | 0.259 | 0.5049 |
| rs55659265 | 2 | 142247852 | A | G | 0.066555 | -0.0244268 | 0.0043179 | 1.50E-08 | 0.35 | 0.1543 | 0.574 |
| rs56306056 | 2 | 184416610 | A | G | 0.214739 | 0.0160763 | 0.00262104 | 8.60E-10 | 0.34 | 0.9399 | 0.9145 |
| rs56392241 | 3 | 131968209 | C | A | 0.393183 | -0.0135943 | 0.0022162 | 8.60E-10 | 0.84 | 0.2337 | 0.0289801 |
| rs56393977 | 18 | 39265197 | T | G | 0.102491 | 0.022399 | 0.00357203 | 3.60E-10 | 0.33 | 0.0114201 | 0.608001 |
| rs57537843 | 2 | 22558973 | A | G | 0.381196 | -0.0160011 | 0.00222247 | 6.00E-13 | 0.2 | 0.0981093 | 0.9346 |
| rs6011138 | 20 | 62440700 | C | T | 0.13593 | 0.0188511 | 0.00315668 | 2.30E-09 | 0.22 | 0.00494402 | 0.8772 |
| rs61746970 | 19 | 51132746 | A | G | 0.039278 | -0.0316935 | 0.00568006 | 2.40E-08 | 0.46 | 0.698601 | 0.0170699 |
| rs61864459 | 10 | 120013564 | T | G | 0.169827 | 0.0161278 | 0.00287967 | 2.10E-08 | 0.43 | 0.5141 | 0.9679 |
| rs62134195 | 2 | 45062249 | T | C | 0.040972 | 0.033951 | 0.00550485 | 6.90E-10 | 0.86 | 0.6313 | 0.3927 |
| rs62370848 | 5 | 124228322 | C | A | 0.198707 | 0.0170776 | 0.00272348 | 3.60E-10 | 0.97 | 0.02175 | 0.3628 |
| rs62439690 | 7 | 21417556 | A | G | 0.262405 | -0.0162081 | 0.00247933 | 6.30E-11 | 0.15 | 0.4236 | 0.1798 |
| rs6486065 | 11 | 12870969 | G | T | 0.708967 | 0.0161936 | 0.00237994 | 1.00E-11 | 0.57 | 0.0905899 | 4.09E-05 |
| rs6517512 | 21 | 40512129 | G | A | 0.955633 | -0.029508 | 0.00524367 | 1.80E-08 | 0.55 | 0.1523 | 0.374 |
| rs6549670 | 3 | 74936132 | G | A | 0.83866 | -0.0182814 | 0.00293516 | 4.70E-10 | 0.34 | 0.1096 | 0.252 |
| rs6564268 | 16 | 75606878 | G | C | 0.056059 | 0.0283611 | 0.00469506 | 1.50E-09 | 0.450001 | 0.1058 | 0.6297 |
| rs6586405 | 1 | 234739101 | A | C | 0.326953 | -0.0130845 | 0.00231009 | 1.50E-08 | 0.24 | 0.2133 | 0.0236903 |
| rs66906321 | 2 | 630070 | C | T | 0.818851 | -0.0181218 | 0.00284922 | 2.00E-10 | 0.0719996 | 0.0234898 | 0.0449304 |
| rs6692613 | 1 | 7524974 | T | C | 0.530087 | -0.012697 | 0.00216093 | 4.20E-09 | 0.0879995 | 0.2109 | 0.1114 |
| rs6719762 | 2 | 60166832 | C | T | 0.473304 | -0.0233857 | 0.00216422 | 3.20E-27 | 0.11 | 0.2553 | 0.1505 |
| rs6744794 | 2 | 44842145 | G | C | 0.623147 | -0.0203928 | 0.00222121 | 4.30E-20 | 0.13 | 0.6842 | 0.0405098 |
| rs6748341 | 2 | 225377574 | G | C | 0.316337 | 0.0167655 | 0.00232362 | 5.40E-13 | 0.31 | 0.00971807 | 0.12 |
| rs6763967 | 3 | 60884616 | A | G | 0.264904 | -0.0140945 | 0.00244253 | 7.90E-09 | 0.27 | 0.7511 | 0.5497 |
| rs67723420 | 3 | 35775115 | A | T | 0.376012 | 0.0145464 | 0.00223412 | 7.50E-11 | 0.52 | 0.9422 | 0.2177 |
| rs6939048 | 6 | 26327953 | A | G | 0.627277 | 0.0143527 | 0.00223278 | 1.30E-10 | 0.33 | 0.0143001 | 0.00897408 |
| rs6955073 | 7 | 118298293 | A | T | 0.508354 | 0.0124183 | 0.00216596 | 9.80E-09 | * | * | * |
| rs6966769 | 7 | 1299334 | G | A | 0.11444 | 0.0185136 | 0.00339345 | 4.90E-08 | 0.9 | 0.00860003 | 0.9539 |
| rs6966898 | 7 | 135221170 | T | C | 0.333033 | -0.0138863 | 0.00230233 | 1.60E-09 | 0.0530005 | 0.02934 | 0.0201999 |
| rs6973256 | 7 | 133055603 | T | C | 0.59757 | 0.0141037 | 0.00221898 | 2.10E-10 | 0.8 | 0.00092651 | 0.8333 |
| rs6978112 | 7 | 1966841 | T | C | 0.411515 | -0.0170735 | 0.00219534 | 7.40E-15 | 0.0179999 | 7.35E-05 | 0.0576501 |
| rs702 | 4 | 28710551 | T | A | 0.838619 | 0.0205228 | 0.00293386 | 2.60E-12 | 0.74 | 0.2498 | 0.3309 |
| rs7025089 | 9 | 134881443 | A | C | 0.680645 | 0.0160854 | 0.00231339 | 3.60E-12 | 0.0280001 | 0.7113 | 0.9697 |
| rs705240 | 3 | 118457615 | T | C | 0.184196 | -0.0160808 | 0.00277714 | 7.00E-09 | 0.22 | 0.1334 | 0.9471 |
| rs7085104 | 10 | 104628873 | G | A | 0.328663 | -0.0146038 | 0.00229572 | 2.00E-10 | 0.36 | 0.1086 | 0.1812 |
| rs710289 | 14 | 98535256 | G | A | 0.415982 | -0.0159545 | 0.00218989 | 3.20E-13 | 0.15 | 0.7031 | 0.1795 |
| rs71433405 | 13 | 97130945 | T | C | 0.066007 | 0.0261044 | 0.00440193 | 3.00E-09 | 0.88 | 0.3418 | 0.3292 |
| rs714393 | 2 | 212698718 | T | C | 0.456816 | 0.0141564 | 0.00216389 | 6.10E-11 | 0.38 | 0.1439 | 0.490999 |
| rs7156339 | 14 | 103366698 | T | C | 0.163624 | -0.0236699 | 0.00291707 | 4.90E-16 | 0.760001 | 0.00400396 | 0.9135 |
| rs7167444 | 15 | 97495941 | T | G | 0.253197 | -0.0141521 | 0.00251088 | 1.70E-08 | 0.96 | 0.3819 | 0.9547 |
| rs7188873 | 16 | 24727064 | G | A | 0.622795 | -0.0172874 | 0.00222805 | 8.60E-15 | 0.0519996 | 0.4409 | 0.0818408 |
| rs7236339 | 18 | 77579773 | A | G | 0.22753 | -0.0204944 | 0.00259028 | 2.50E-15 | 0.91 | 0.4912 | 0.749 |
| rs72674824 | 8 | 95489281 | C | T | 0.24179 | 0.0147127 | 0.002525 | 5.60E-09 | 0.96 | 0.9099 | 0.9493 |
| rs72822625 | 5 | 167757575 | A | G | 0.094583 | 0.0215117 | 0.00368811 | 5.50E-09 | 0.780001 | 0.0840195 | 0.0152999 |
| rs72887338 | 6 | 67536056 | C | T | 0.386847 | -0.0164161 | 0.0022168 | 1.30E-13 | 0.94 | # | 0.757 |
| rs72996415 | 6 | 105265993 | C | T | 0.064661 | 0.0254579 | 0.0044961 | 1.50E-08 | 0.52 | 0.9761 | 0.1374 |
| rs7381195 | 5 | 60030791 | A | T | 0.611957 | -0.014635 | 0.00221414 | 3.80E-11 | 0.82 | 0.5401 | 0.517399 |
| rs74583305 | 8 | 143419130 | T | C | 0.020359 | 0.0437545 | 0.00766429 | 1.10E-08 | 0.27 | 0.0849493 | 0.6316 |
| rs7476 | 11 | 46342834 | C | A | 0.312212 | -0.0139275 | 0.00233764 | 2.60E-09 | 0.19 | 0.9275 | 0.00961391 |
| rs75082770 | 2 | 185915324 | G | A | 0.037667 | -0.0352385 | 0.00569853 | 6.30E-10 | 0.52 | 0.8913 | 0.2522 |
| rs75783371 | 13 | 28109961 | C | T | 0.139303 | 0.018662 | 0.00315196 | 3.20E-09 | 0.83 | 0.8153 | 0.636099 |
| rs76513770 | 16 | 72505534 | C | T | 0.127724 | 0.0256106 | 0.00323446 | 2.40E-15 | 0.46 | 0.0520295 | 0.4146 |
| rs767943 | 6 | 23446691 | A | C | 0.264898 | -0.0186374 | 0.00246006 | 3.60E-14 | 0.35 | 0.0288802 | 0.4014 |
| rs77214504 | 1 | 75316394 | T | A | 0.047379 | 0.0348552 | 0.00508475 | 7.10E-12 | 0.57 | 0.02082 | 0.0510305 |
| rs7729019 | 5 | 155844242 | T | C | 0.613575 | 0.0126365 | 0.0022139 | 1.10E-08 | 0.75 | 0.1832 | 0.579201 |
| rs7785195 | 7 | 3424686 | A | G | 0.659377 | 0.0158783 | 0.00227544 | 3.00E-12 | 0.1 | 0.2036 | 0.0771703 |
| rs7804551 | 7 | 99119110 | G | A | 0.15369 | 0.0208069 | 0.00298824 | 3.30E-12 | 0.11 | 0.3261 | 0.7272 |
| rs7815125 | 8 | 87680112 | A | T | 0.826612 | 0.0187851 | 0.00284902 | 4.30E-11 | 0.68 | 0.7822 | 0.9056 |
| rs783544 | 15 | 83240293 | C | A | 0.750616 | 0.0164657 | 0.00249373 | 4.00E-11 | 0.67 | 0.09683 | 0.3783 |
| rs784255 | 18 | 53403228 | T | G | 0.475487 | -0.0142825 | 0.00216716 | 4.40E-11 | 0.23 | 0.483 | 0.3808 |
| rs7857266 | 9 | 96381765 | T | C | 0.395169 | 0.0131398 | 0.00222736 | 3.70E-09 | 0.15 | 0.2239 | 0.0270097 |
| rs7868984 | 9 | 23357826 | C | T | 0.415886 | 0.0138367 | 0.00219061 | 2.70E-10 | 0.96 | 0.5512 | 0.1214 |
| rs7909331 | 10 | 11205224 | G | A | 0.164505 | -0.0167017 | 0.00290726 | 9.20E-09 | 0.0239999 | 0.0945998 | 0.9235 |
| rs7911962 | 10 | 9968483 | T | C | 0.384406 | 0.0135852 | 0.00222867 | 1.10E-09 | 0.68 | 0.2946 | 0.7987 |
| rs79231171 | 2 | 213841704 | A | C | 0.077097 | 0.0224426 | 0.00403721 | 2.70E-08 | 0.86 | 0.3601 | 0.842 |
| rs79269403 | 3 | 108036819 | A | G | 0.230853 | 0.0202286 | 0.00256984 | 3.50E-15 | 0.0599998 | 0.0904191 | 0.00903296 |
| rs794375 | 7 | 75147801 | C | T | 0.42645 | 0.0155464 | 0.00219208 | 1.30E-12 | 0.38 | 0.0120199 | 0.9034 |
| rs8003519 | 14 | 93826672 | G | A | 0.581483 | -0.0149416 | 0.00219233 | 9.40E-12 | 0.83 | 0.06531 | 0.5751 |
| rs800532 | 8 | 116842110 | G | A | 0.776043 | -0.0156462 | 0.00258941 | 1.50E-09 | 0.61 | 0.0476596 | 0.1149 |
| rs803679 | 1 | 44349405 | A | G | 0.792617 | 0.0197133 | 0.0026525 | 1.10E-13 | 0.96 | 0.64 | 0.2534 |
| rs807478 | 19 | 36252494 | G | A | 0.497201 | 0.0142362 | 0.00216259 | 4.60E-11 | 0.9 | 0.7008 | 0.142 |
| rs8096225 | 18 | 36921851 | C | A | 0.694761 | -0.0132106 | 0.00234688 | 1.80E-08 | 0.33 | 0.1055 | 0.628599 |
| rs8133065 | 21 | 31422507 | G | C | 0.283251 | 0.013895 | 0.00241406 | 8.60E-09 | 0.39 | 0.9188 | 0.0227997 |
| rs838039 | 2 | 140342288 | A | G | 0.686798 | 0.0147669 | 0.00232368 | 2.10E-10 | 0.450001 | 0.5602 | 0.552199 |
| rs9491228 | 6 | 125061384 | C | T | 0.528945 | -0.0141344 | 0.00216535 | 6.70E-11 | 0.79 | 0.547599 | 0.1383 |
| rs9514600 | 13 | 107644422 | G | C | 0.502126 | -0.0119302 | 0.00216113 | 3.40E-08 | * | * | * |
| rs9536961 | 13 | 55678332 | G | A | 0.348212 | 0.0130856 | 0.00229334 | 1.20E-08 | 0.8 | 0.9794 | 0.4399 |
| rs9538248 | 13 | 59492828 | A | C | 0.320928 | -0.0156218 | 0.00230917 | 1.30E-11 | 0.98 | 0.01554 | 0.226 |
| rs976179 | 2 | 198880378 | T | A | 0.485183 | -0.0119687 | 0.00215899 | 3.00E-08 | * | * | * |
| rs9809849 | 3 | 3726156 | A | G | 0.425234 | -0.0141765 | 0.0021849 | 8.70E-11 | 0.25 | 0.0875992 | 0.4277 |
| rs9866968 | 3 | 85683470 | A | G | 0.349391 | 0.0244328 | 0.00227411 | 6.30E-27 | 0.92 | 0.3272 | 0.2711 |
| rs9873182 | 3 | 88250597 | G | C | 0.841159 | -0.0257397 | 0.00294454 | 2.30E-18 | 0.62 | 0.2096 | 0.5675 |
| rs9886840 | 9 | 124602728 | G | A | 0.580117 | 0.0125283 | 0.0021869 | 1.00E-08 | 0.38 | 0.0932202 | 0.7637 |
| rs9891146 | 17 | 65988049 | C | T | 0.734943 | 0.0142035 | 0.00244483 | 6.30E-09 | 0.91 | 0.00693697 | 0.0553095 |
| rs9904818 | 17 | 77793292 | C | G | 0.090226 | 0.0223466 | 0.00377862 | 3.30E-09 | 0.0439997 | 0.1088 | 0.0701294 |
| rs9923553 | 16 | 5825579 | G | A | 0.290128 | -0.0143033 | 0.00237602 | 1.70E-09 | 0.35 | 0.4529 | 0.0993002 |
| rs993700 | 4 | 67825894 | C | T | 0.77749 | 0.0162142 | 0.00258969 | 3.80E-10 | 0.15 | 0.7698 | 0.3665 |
| rs9964201 | 18 | 50600552 | A | C | 0.082656 | 0.0264901 | 0.00392756 | 1.50E-11 | 0.719999 | 0.3629 | 0.6463 |
| Number of SNPs | | | | | | | | | 190 | 189 | 190 |
| F-statistics | | | | | | | | | 46.12 | 46.06 | 46.12 |
| SNP | Chr | Position | Effect allele | Other allele | EAF | Exposure (ASOC) | | | Outcome (P value) | | |
|  |  |  |  |  |  | Beta | SE | P value | Overall OA | Knee OA | Hip OA |
| rs12543077 | 8 | 50943469 | T | C | 0.586248 | 0.018042 | 0.00321596 | 2.00E-08 | 0.91 | 0.5426 | 0.4713 |
| rs12739102 | 1 | 190693637 | G | T | 0.037744 | 0.0463634 | 0.00846141 | 4.30E-08 | 0.709999 | 0.3003 | 0.00524602 |
| rs13066140 | 3 | 50154223 | C | T | 0.112513 | 0.0279243 | 0.00501536 | 2.60E-08 | 0.22 | 0.0470002 | 0.0579002 |
| rs72636642 | 4 | 67942411 | C | A | 0.120375 | -0.0292879 | 0.00487675 | 1.90E-09 | 0.19 | 0.0290102 | 0.7295 |
| No.of SNPs | | | | | | | | | 4 | 4 | 4 |
| F-statistics | | | | | | | | | 32.16 | 32.16 | 32.16 |

Note: The information in this table is for all SNPs that have undergone initial selecting (limiting the *P* value as 5×10^−8^ and removing the interference of linkage disequilibrium). * : Excluded SNPs with incompatible alleles or palindromic structure after harmonizing to OA, #: SNPs that were not adopted by initially screening in present type OA.
