# Supplementary material for: Causal association of menstrual reproductive factors on the risk of osteoarthritis: A univariate and multivariate Mendelian randomization study
Source: PLoS One. 2024 Aug 30;19(8):e0307958. doi: 10.1371/journal.pone.0307958 (PMC11364240; doi:10.1371/journal.pone.0307958)
Supplement: S2 Table — (DOCX) [file pone.0307958.s002.docx]

**Supplementary Table 2：Univariable Mendelian randomization estimates results for menstrual reproductive factors on osteoarthritis.**

| Exposure | Outcome | No. of SNPs | MR results | | | | |
| --- | --- | --- | --- | --- | --- | --- | --- |
|  |  |  | Method | Beta | SE | P value | OR (95%CI) |
| NLB | KOA | 3 | MR Egger | 1.0872 | 1.6699 | 6.33E-01 | 2.9660 (0.1124, 78.2699) |
| NLB | KOA | 3 | Weighted median | -0.2519 | 0.2555 | 3.24E-01 | 0.7773 (0.4712, 1.2825) |
| NLB | KOA | 3 | Inverse variance weighted | -0.2206 | 0.2294 | 3.36E-01 | 0.8020 (0.5115, 1.2575) |
| NLB | HOA | 3 | MR Egger | 3.3779 | 2.1003 | 3.54E-01 | 29.3079 (0.4778, 1797.8702) |
| NLB | HOA | 3 | Weighted median | -0.1240 | 0.3601 | 7.31E-01 | 0.8834 (0.4361, 1.7894) |
| NLB | HOA | 3 | Inverse variance weighted | -0.2482 | 0.3557 | 4.85E-01 | 0.7802 (0.3886, 1.5667) |
| NLB | OOA | 3 | MR Egger | -0.0037 | 0.1359 | 9.82E-01 | 0.9963 (0.7634, 1.3002) |
| NLB | OOA | 3 | Weighted median | 0.0351 | 0.0175 | 4.47E-02 | 1.0357 (1.0008, 1.0718) |
| NLB | OOA | 3 | Inverse variance weighted | 0.0310 | 0.0136 | 2.28E-02 | 1.0314 (1.0043, 1.0593) |
| AFB | KOA | 17 | MR Egger | -0.8179 | 0.6485 | 2.27E-01 | 0.4414 (0.1238, 1.5734) |
| AFB | KOA | 17 | Weighted median | -0.5015 | 0.1436 | 4.79E-04 | 0.6056 (0.4570, 0.8025) |
| AFB | KOA | 17 | Inverse variance weighted | -0.5296 | 0.1200 | 1.02E-05 | 0.5888 (0.4654, 0.7450) |
| AFB | HOA | 17 | MR Egger | -0.2957 | 0.8771 | 7.41E-01 | 0.7440 (0.1333, 4.1513) |
| AFB | HOA | 17 | Weighted median | -0.1821 | 0.1838 | 3.22E-01 | 0.8335 (0.5813, 1.1951) |
| AFB | HOA | 17 | Inverse variance weighted | -0.2596 | 0.1611 | 1.07E-01 | 0.7714 (0.5625, 1.0579) |
| AFB | OOA | 17 | MR Egger | -0.0424 | 0.0368 | 2.68E-01 | 0.9585 (0.8917, 1.0303) |
| AFB | OOA | 17 | Weighted median | -0.0210 | 0.0089 | 1.78E-02 | 0.9792 (0.9624, 0.9964) |
| AFB | OOA | 17 | Inverse variance weighted | -0.0171 | 0.0068 | 1.24E-02 | 0.9830 (0.9699, 0.9963) |
| AMP | KOA | 109 | MR Egger | 0.0229 | 0.0708 | 7.47E-01 | 1.0231 (0.8906, 1.1754) |
| AMP | KOA | 109 | Weighted median | -0.0027 | 0.0425 | 9.49E-01 | 0.9973 (0.9176, 1.0839) |
| AMP | KOA | 109 | Inverse variance weighted | 0.0179 | 0.0348 | 6.06E-01 | 1.0181 (0.9510, 1.0899) |
| AMP | HOA | 109 | MR Egger | 0.0259 | 0.0750 | 7.31E-01 | 1.0262 (0.8860, 1.1887) |
| AMP | HOA | 109 | Weighted median | 0.0606 | 0.0534 | 2.56E-01 | 1.0624 (0.9569, 1.1796) |
| AMP | HOA | 109 | Inverse variance weighted | 0.0965 | 0.0371 | 9.33E-03 | 1.1013 (1.0240, 1.1844) |
| AMP | OOA | 108 | MR Egger | 0.0035 | 0.0037 | 3.43E-01 | 1.0035 (0.9963, 1.0109) |
| AMP | OOA | 108 | Weighted median | 0.0007 | 0.0025 | 7.65E-01 | 1.0007 (0.9959, 1.0056) |
| AMP | OOA | 108 | Inverse variance weighted | 0.0003 | 0.0018 | 8.67E-01 | 1.0003 (0.9967, 1.0039) |
| AAM | KOA | 194 | MR Egger | -0.1506 | 0.1482 | 3.11E-01 | 0.8602 (0.6433, 1.1501) |
| AAM | KOA | 194 | Weighted median | -0.0979 | 0.0722 | 1.75E-01 | 0.9067 (0.7871, 1.0446) |
| AAM | KOA | 194 | Inverse variance weighted | -0.1982 | 0.0534 | 2.08E-04 | 0.8202 (0.7386, 0.9108) |
| AAM | HOA | 193 | MR Egger | 0.0865 | 0.2097 | 6.81E-01 | 1.0903 (0.7229, 1.6445) |
| AAM | HOA | 193 | Weighted median | -0.0119 | 0.0860 | 8.90E-01 | 0.9882 (0.8348, 1.1697) |
| AAM | HOA | 193 | Inverse variance weighted | -0.0434 | 0.0756 | 5.66E-01 | 0.9576 (0.8258, 1.1104) |
| AAM | OOA | 194 | MR Egger | 0.0043 | 0.0075 | 5.65E-01 | 1.0043 (0.9896, 1.0193) |
| AAM | OOA | 194 | Weighted median | 0.0003 | 0.0043 | 9.44E-01 | 1.0003 (0.9919, 1.0088) |
| AAM | OOA | 194 | Inverse variance weighted | -0.0035 | 0.0027 | 1.94E-01 | 0.9965 (0.9912, 1.0018) |
| AFSI | KOA | 189 | MR Egger | -0.4458 | 0.2739 | 1.05E-01 | 0.6403 (0.3744, 1.0953) |
| AFSI | KOA | 189 | Weighted median | -0.5261 | 0.0736 | 8.58E-13 | 0.5909 (0.5116, 0.6826) |
| AFSI | KOA | 189 | Inverse variance weighted | -0.5993 | 0.0606 | 4.31E-23 | 0.5492 (0.4877, 0.6184) |
| AFSI | HOA | 190 | MR Egger | -0.2561 | 0.3269 | 4.34E-01 | 0.7741 (0.4078, 1.4692) |
| AFSI | HOA | 190 | Weighted median | -0.1792 | 0.0916 | 5.05E-02 | 0.8359 (0.6985, 1.0004) |
| AFSI | HOA | 190 | Inverse variance weighted | -0.1660 | 0.0720 | 2.11E-02 | 0.8470 (0.7355, 0.9754) |
| AFSI | OOA | 190 | MR Egger | -0.0027 | 0.0135 | 8.41E-01 | 0.9973 (0.9713, 1.0240) |
| AFSI | OOA | 190 | Weighted median | -0.0152 | 0.0042 | 3.05E-04 | 0.9849 (0.9768, 0.9931) |
| AFSI | OOA | 190 | Inverse variance weighted | -0.0147 | 0.0030 | 7.35E-07 | 0.9854 (0.9797, 0.9912) |
| ALB | KOA | 6 | MR Egger | -0.9467 | 0.7374 | 2.69E-01 | 0.3880 (0.0915, 1.6464) |
| ALB | KOA | 6 | Weighted median | -0.6762 | 0.2520 | 7.30E-03 | 0.5085 (0.3103, 0.8334) |
| ALB | KOA | 6 | Inverse variance weighted | -0.7632 | 0.1994 | 1.29E-04 | 0.4662 (0.3154, 0.6891) |
| ALB | HOA | 6 | MR Egger | -0.6655 | 1.0216 | 5.50E-01 | 0.5140 (0.0694, 3.8071) |
| ALB | HOA | 6 | Weighted median | 0.3424 | 0.3401 | 3.14E-01 | 1.4083 (0.7231, 2.7429) |
| ALB | HOA | 6 | Inverse variance weighted | 0.0150 | 0.2877 | 9.59E-01 | 1.0151 (0.5775, 1.7842) |
| ALB | OOA | 6 | MR Egger | 0.0169 | 0.0323 | 6.29E-01 | 1.0170 (0.9546, 1.0835) |
| ALB | OOA | 6 | Weighted median | -0.0252 | 0.0140 | 7.24E-02 | 0.9751 (0.9487, 1.0023) |
| ALB | OOA | 6 | Inverse variance weighted | -0.0214 | 0.0103 | 3.77E-02 | 0.9789 (0.9593, 0.9988) |
| ASOC | KOA | 4 | MR Egger | -1.7820 | 0.9601 | 2.05E-01 | 0.1683 (0.0256, 1.1050) |
| ASOC | KOA | 4 | Weighted median | -0.8082 | 0.3297 | 1.42E-02 | 0.4456 (0.2335, 0.8505) |
| ASOC | KOA | 4 | Inverse variance weighted | -0.6063 | 0.3246 | 6.17E-02 | 0.5453 (0.2887, 1.0303) |
| ASOC | HOA | 4 | MR Egger | 2.1563 | 2.0242 | 3.98E-01 | 8.6395 (0.1635, 456.5654) |
| ASOC | HOA | 4 | Weighted median | -0.3490 | 0.4221 | 4.08E-01 | 0.7054 (0.3084, 1.6135) |
| ASOC | HOA | 4 | Inverse variance weighted | -0.0379 | 0.6527 | 9.54E-01 | 0.9628 (0.2679, 3.4605) |
| ASOC | OOA | 4 | MR Egger | -0.0409 | 0.0503 | 5.02E-01 | 0.9600 (0.8699, 1.0594) |
| ASOC | OOA | 4 | Weighted median | -0.0262 | 0.0177 | 1.37E-01 | 0.9741 (0.9410, 1.0084) |
| ASOC | OOA | 4 | Inverse variance weighted | -0.0236 | 0.0154 | 1.26E-01 | 0.9767 (0.9476, 1.0067) |

Note: NLB, Number of live births; AFB, Age at first live birth; AMP, Age at menopause; AAM, Age at menarche; AFSI, Age first had sexual intercourse; ALB, Age at last live birth; ASOC, Age started oral contraceptive pill.
