# Supplementary material for: Causal association of menstrual reproductive factors on the risk of osteoarthritis: A univariate and multivariate Mendelian randomization study
Source: PLoS One. 2024 Aug 30;19(8):e0307958. doi: 10.1371/journal.pone.0307958 (PMC11364240; doi:10.1371/journal.pone.0307958)
Supplement: S3 Table — (DOCX) [file pone.0307958.s003.docx]

**Supplementary Table 3: multivariate Mendelian randomization estimates results for menstrual reproductive factors on osteoarthritis.**

| Exposure | Outcome | No. of SNPs | Beta | SE | P value | OR (95%CI) |
| --- | --- | --- | --- | --- | --- | --- |
| BMI | OOA | 127 | 0.0080 | 0.0036 | 2.61E-02 | 1.0080 (1.0009, 1.0151) |
| AAM | OOA | 60 | 0.0058 | 0.0048 | 2.31E-01 | 1.0058 (0.9963, 1.0153) |
| BMI | KOA | 127 | 0.7139 | 0.0581 | 9.46E-35 | 2.0420 (1.8224, 2.2881) |
| AAM | KOA | 59 | 0.0601 | 0.0786 | 4.44E-01 | 1.0619 (0.9104, 1.2388) |
| BMI | HOA | 126 | 0.5985 | 0.0804 | 9.79E-14 | 1.8194 (1.5541, 2.1299) |
| AAM | HOA | 59 | 0.1895 | 0.1080 | 7.93E-02 | 1.2087 (0.9781, 1.4937) |
| BMI | OOA | 138 | 0.0073 | 0.0031 | 1.86E-02 | 1.0074 (1.0012, 1.0135) |
| AMP | OOA | 35 | 0.0015 | 0.0025 | 5.57E-01 | 1.0015 (0.9966, 1.0063) |
| BMI | KOA | 138 | 0.7493 | 0.0488 | 3.78E-53 | 2.1155 (1.9225, 2.3280) |
| AMP | KOA | 35 | 0.0206 | 0.0387 | 5.95E-01 | 1.0208 (0.9462, 1.1013) |
| BMI | HOA | 137 | 0.4875 | 0.0652 | 7.25E-14 | 1.6283 (1.4331, 1.8501) |
| AMP | HOA | 35 | 0.1093 | 0.0514 | 3.34E-02 | 1.1155 (1.0086, 1.2337) |
| BMI | OOA | 171 | -0.0018 | 0.0036 | 6.19E-01 | 0.9982 (0.9911, 1.0053) |
| AFB | OOA | 1 | -0.0302 | 0.0084 | 3.39E-04 | 0.9703 (0.9544, 0.9864) |
| BMI | KOA | 171 | 0.5994 | 0.0564 | 2.27E-26 | 1.8211 (1.6304, 2.0340) |
| AFB | KOA | 1 | -0.5076 | 0.1310 | 1.07E-04 | 0.6019 (0.4656, 0.7782) |
| BMI | HOA | 170 | 0.4584 | 0.0786 | 5.46E-09 | 1.5815 (1.3557, 1.8448) |
| AFB | HOA | 1 | 0.0305 | 0.1831 | 8.68E-01 | 1.0310 (0.7202, 1.4759) |
| BMI | OOA | 171 | 0.0015 | 0.0036 | 6.77E-01 | 1.0015(0.9945, 1.0086) |
| ALB | OOA | 2 | -0.0222 | 0.0102 | 2.98E-02 | 0.9781 (0.9587, 0.9978) |
| BMI | KOA | 171 | 0.6320 | 0.0556 | 6.70E-30 | 1.8814 (1.6870, 2.0982) |
| ALB | KOA | 2 | -0.4897 | 0.1589 | 2.06E-03 | 0.6128 (0.4488, 0.8367) |
| BMI | HOA | 170 | 0.4752 | 0.0762 | 4.39E-10 | 1.6084 (1.3853, 1.8673) |
| ALB | HOA | 2 | 0.1289 | 0.2181 | 5.54E-01 | 1.1376 (0.7419, 1.7443) |
| BMI | OOA | 171 | 0.0045 | 0.0031 | 1.45E-01 | 1.0045 (0.9984, 1.0107) |
| NLB | OOA | 2 | 0.0189 | 0.0098 | 5.46E-02 | 1.0191 (0.9996, 1.0389) |
| BMI | KOA | 171 | 0.7125 | 0.0483 | 2.56E-49 | 2.0390 (1.8550, 2.2414) |
| NLB | KOA | 2 | 0.2394 | 0.1527 | 1.17E-01 | 1.2705 (0.9419, 1.7137) |
| BMI | HOA | 170 | 0.4512 | 0.0654 | 5.08E-12 | 1.5701 (1.3814, 1.7847) |
| NLB | HOA | 2 | 0.0134 | 0.2069 | 9.48E-01 | 1.0135 (0.6756, 1.5203) |
| BMI | OOA | 150 | 0.0016 | 0.0035 | 6.44E-01 | 1.0016 (0.9948, 1.0085) |
| AFSI | OOA | 34 | -0.0213 | 0.0071 | 2.66E-03 | 0.9789 (0.9654, 0.9926) |
| BMI | KOA | 150 | 0.6515 | 0.0552 | 3.45E-32 | 1.9184 (1.7218, 2.1375) |
| AFSI | KOA | 33 | -0.4160 | 0.1133 | 2.42E-04 | 0.6597 (0.5283, 0.8238) |
| BMI | HOA | 149 | 0.4707 | 0.0745 | 2.72E-10 | 1.6011 (1.3834, 1.8530) |
| AFSI | HOA | 33 | 0.0890 | 0.1523 | 5.59E-01 | 1.0931 (0.8110, 1.4733) |
| BMI | OOA | 170 | 0.0036 | 0.0032 | 2.54E-01 | 1.0036 (0.9974, 1.0099) |
| ASOC | OOA | 1 | -0.0266 | 0.0124 | 3.24E-02 | 0.9738 (0.9504, 0.9978) |
| BMI | KOA | 170 | 0.6887 | 0.0489 | 4.00E-45 | 1.9911 (1.8093, 2.1912) |
| ASOC | KOA | 1 | -0.5450 | 0.1918 | 4.49E-03 | 0.5798 (0.3981, 0.8445) |
| BMI | HOA | 169 | 0.4614 | 0.0675 | 7.88E-12 | 1.5864 (1.3899, 1.8106) |
| ASOC | HOA | 1 | 0.1674 | 0.2638 | 5.26E-01 | 1.1822 (0.7049, 1.9827) |

Note: AAM, Age at menarche; AMP, Age at menopause; AFB, Age at first live birth; ALB, Age at last live birth; NLB, Number of live births; AFSI, Age first had sexual intercourse; ASOC, Age started oral contraceptive pill; BMI, Body mass index.
