# Supplementary material for: Causal association of menstrual reproductive factors on the risk of osteoarthritis: A univariate and multivariate Mendelian randomization study
Source: PLoS One. 2024 Aug 30;19(8):e0307958. doi: 10.1371/journal.pone.0307958 (PMC11364240; doi:10.1371/journal.pone.0307958)
Supplement: S2 Fig — (DOCX) [file pone.0307958.s005.docx]

**Supplementary Figure 2:** **Leave-one-out analysis plot of SNPs associated with** **menstrual reproductive factors and osteoarthritis.**

(a)MR estimates for NLB on KOA (b) MR estimates for NLB on HOA (c) MR estimates for NLB on OOA

(d) MR estimates for AFB on KOA (e) MR estimates for AFB on HOA (f) MR estimates for AFB on OOA

(h) MR estimates for ALB on KOA (i) MR estimates for ALB on HOA (j) MR estimates for ALB on OOA

(k) MR estimates for AAM on KOA (l) MR estimates for AAM on HOA (m) MR estimates for AAM on OOA

(n) MR estimates for AMP on KOA (o) MR estimates for AMP on HOA (p) MR estimates for AMP on OOA

(q) MR estimates for AFSI on KOA (r) MR estimates for AFSI on HOA (s) MR estimates for AFSI on OOA

(t) MR estimates for ASOC on KOA (u) MR estimates for ASOC on HOA (v) MR estimates for ASOC on OOA


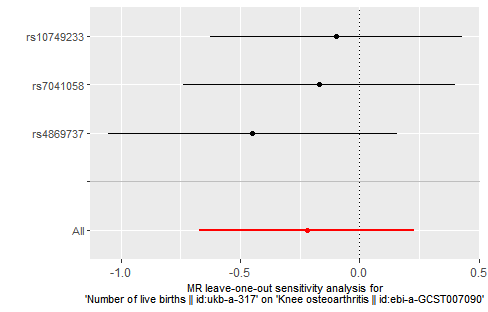

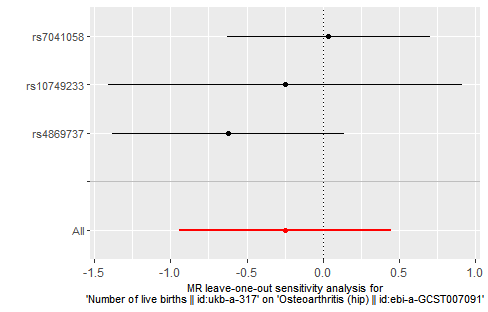

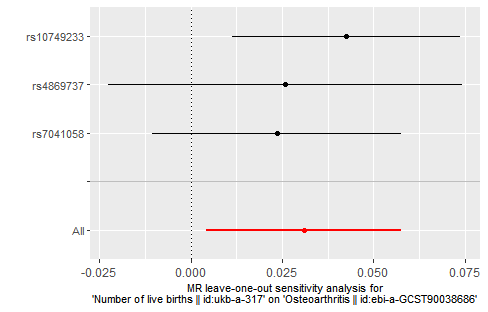


(a) (b) (c)


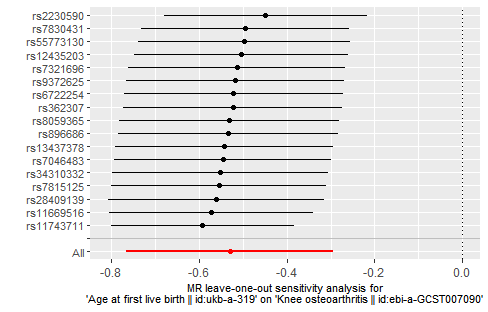

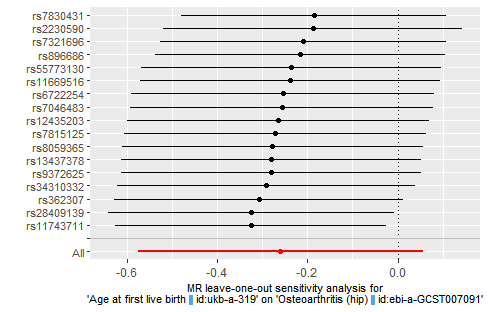

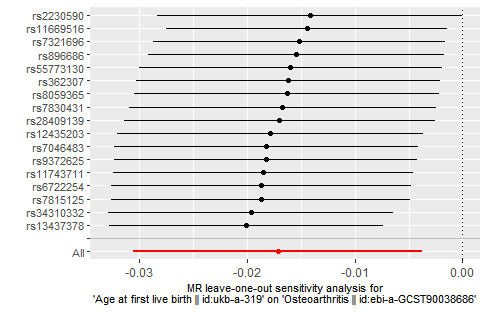


(d) (e) (f)


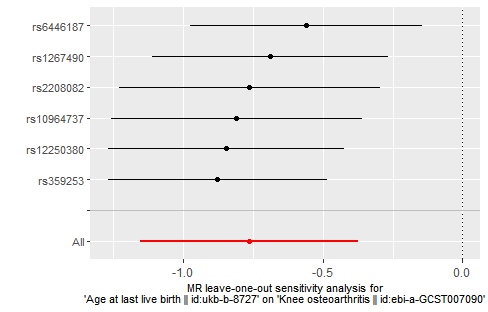

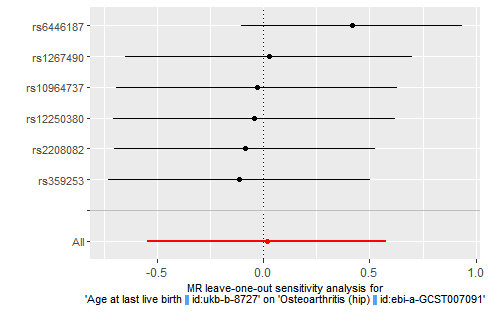

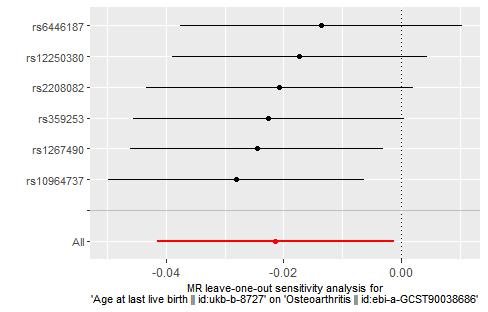


(h) (i) (j)


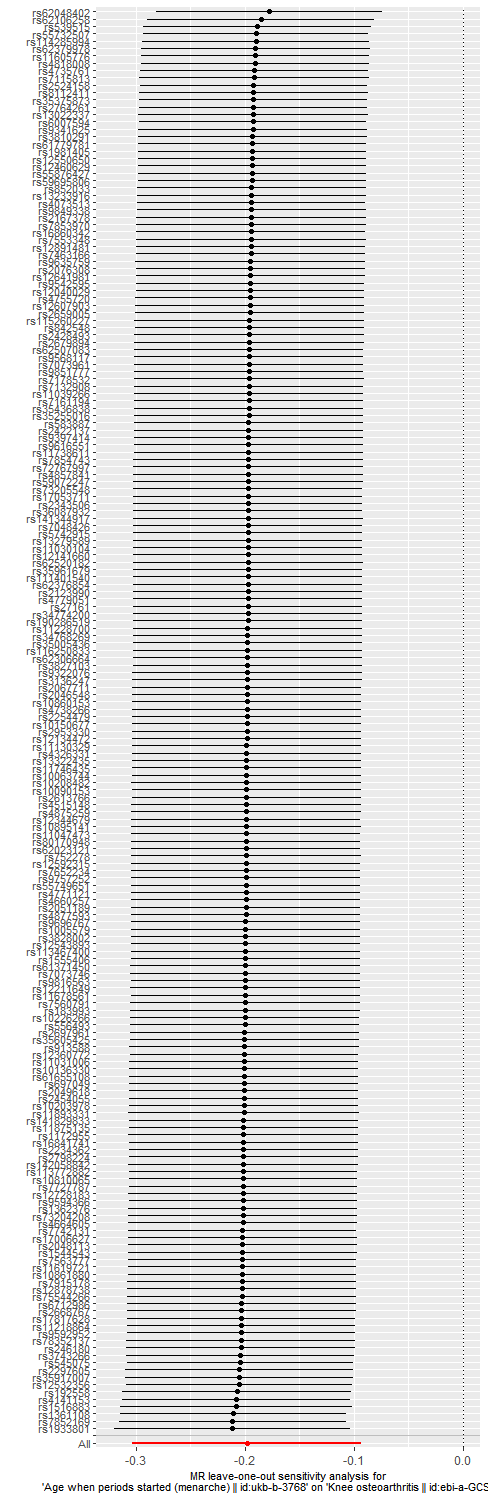

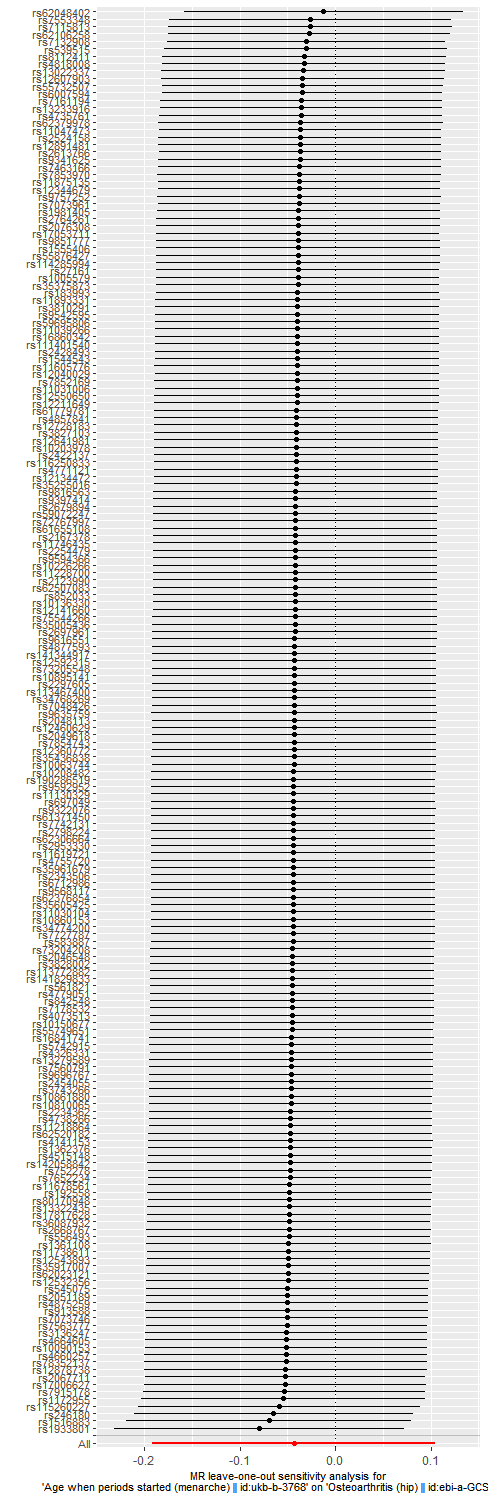

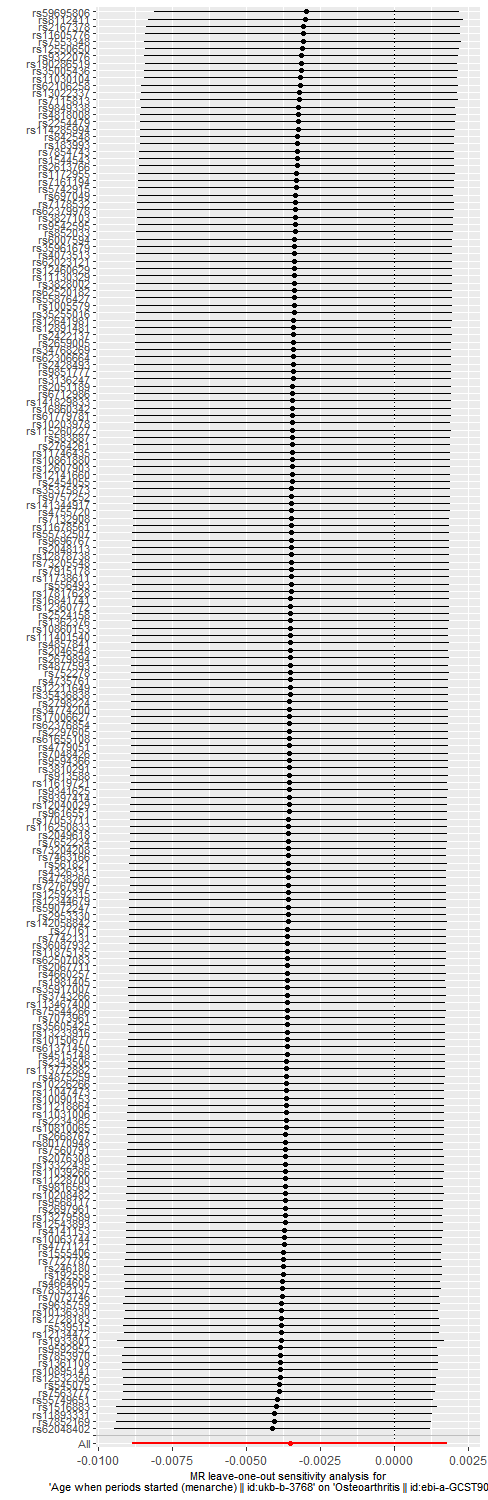


(k) (l) (m)


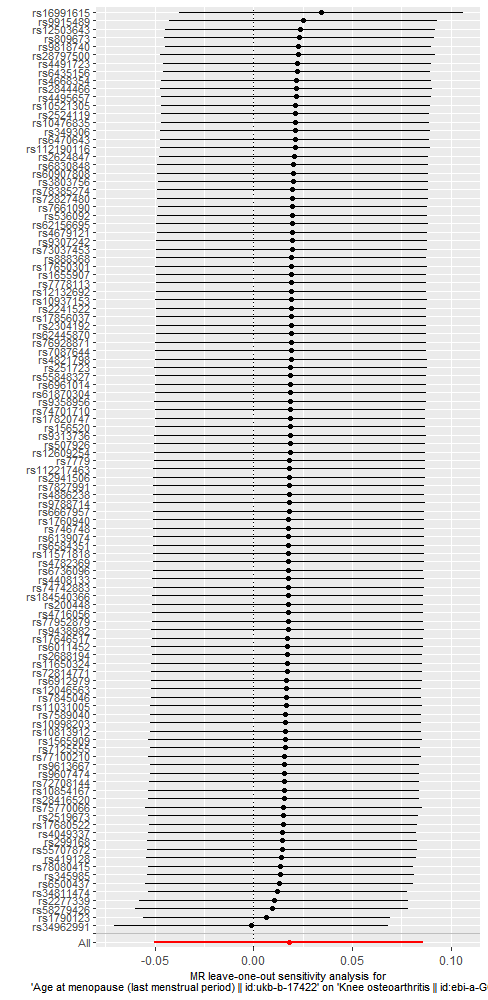

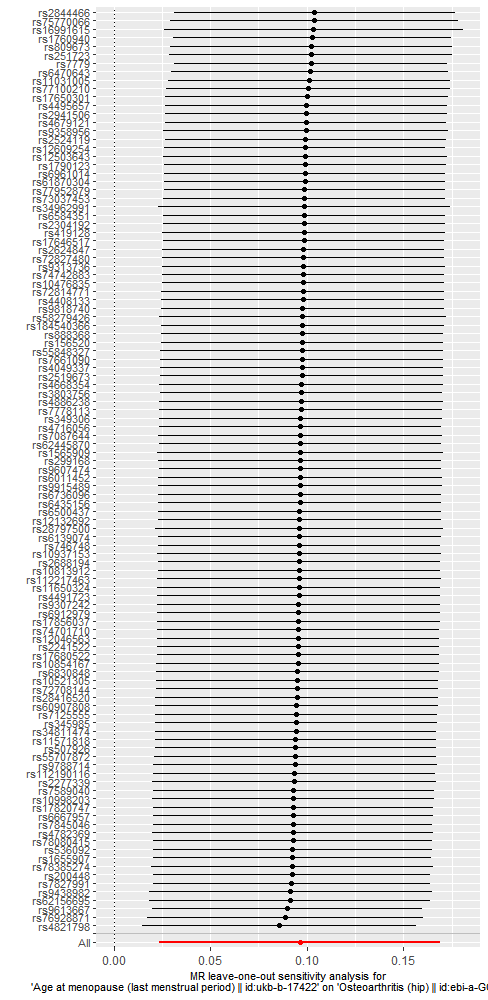

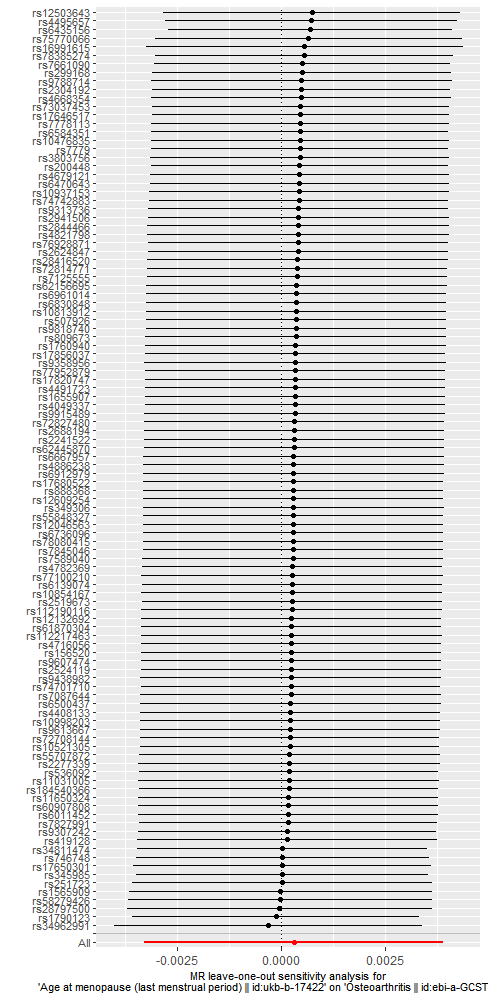


(n) (o) (p)


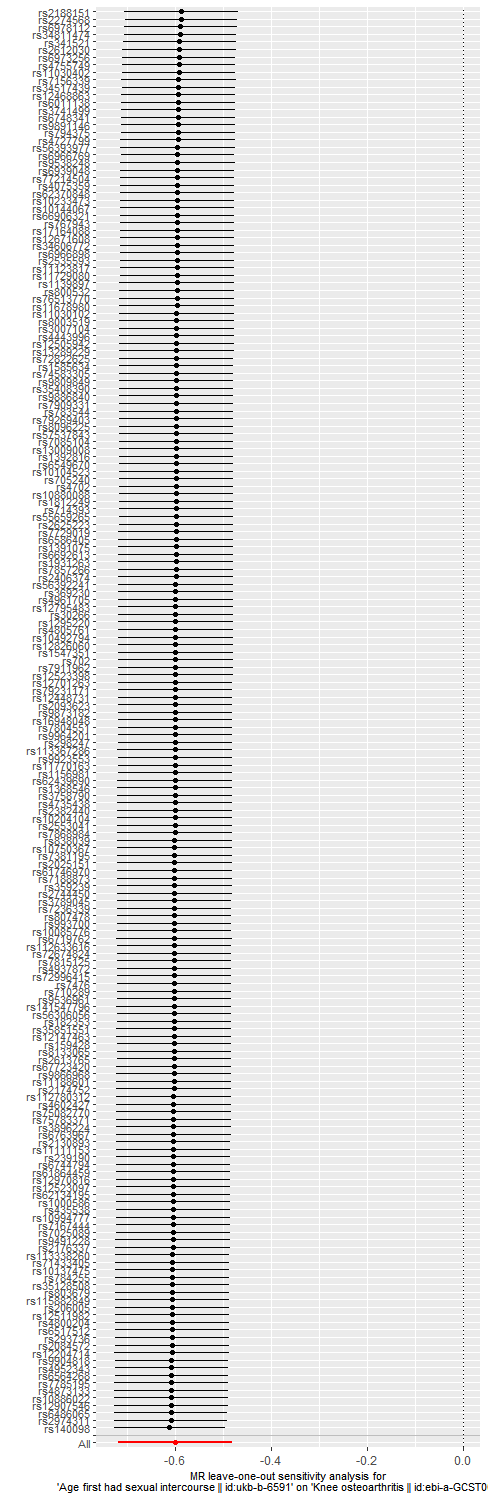

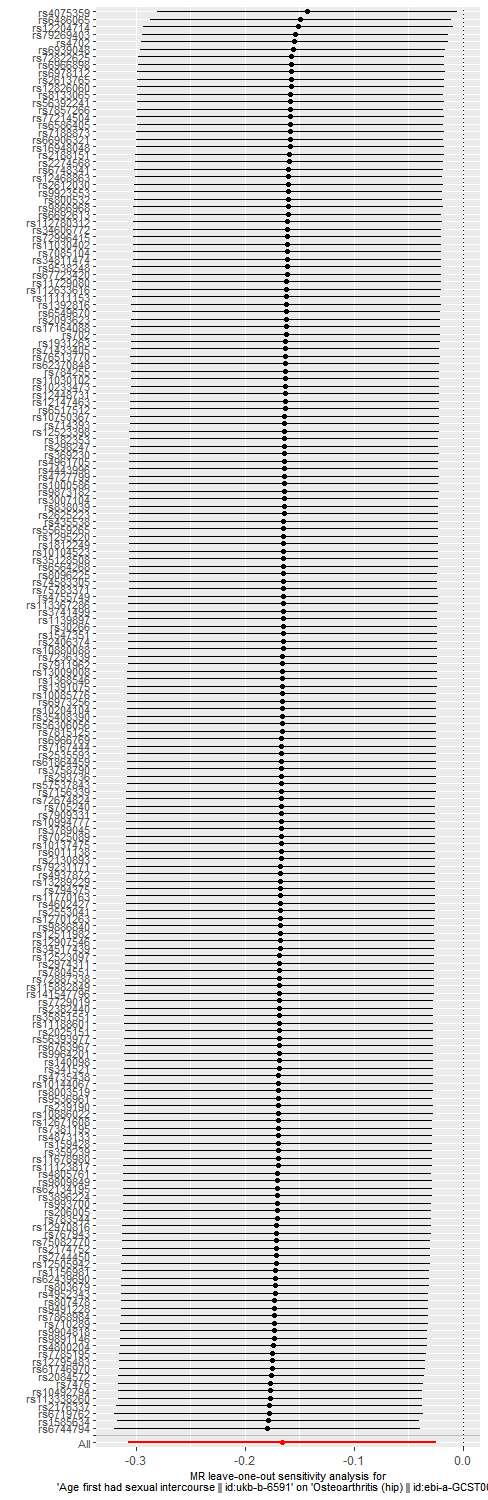

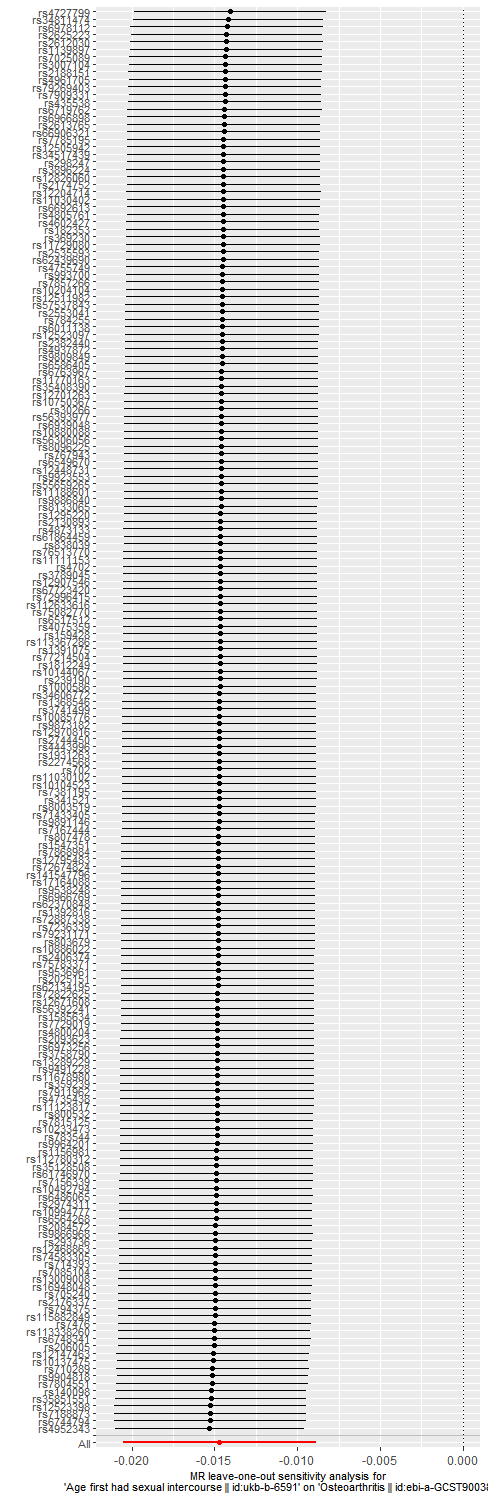


(q) (r) (s)


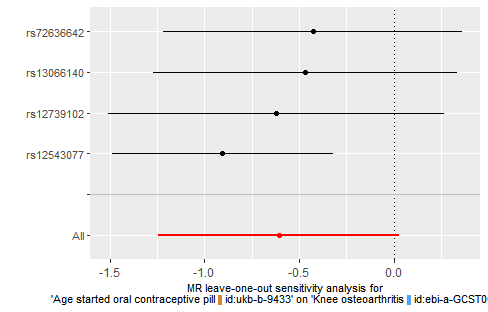

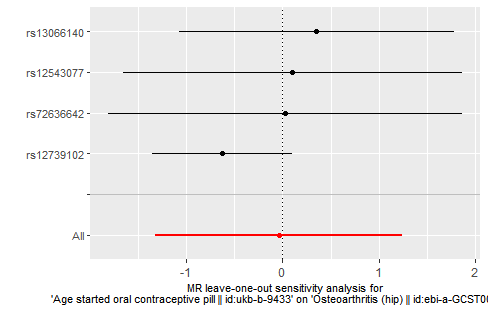

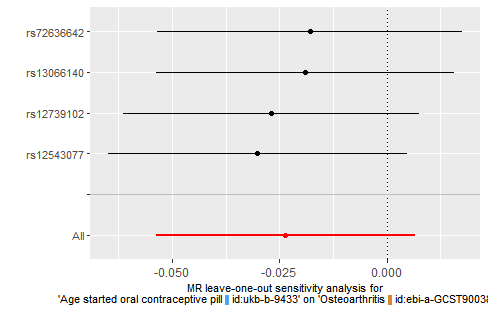


(t) (u) (v)
